# Cooperative activation of PDK1 and AKT by MAPK4 enhances cancer growth and resistance to therapy

**DOI:** 10.1371/journal.pbio.3002227

**Published:** 2023-08-02

**Authors:** Dong Han, Wei Wang, Julie Heejin Jeon, Tao Shen, Xiangsheng Huang, Ping Yi, Bingning Dong, Feng Yang

**Affiliations:** 1 Department of Molecular and Cellular Biology, Baylor College of Medicine, Houston, Texas, United States of America; 2 Center for Nuclear Receptors and Cell Signaling, Department of Biology and Biochemistry, University of Houston, Houston, Texas, United States of America; 3 Department of Medicine, Baylor College of Medicine, Houston, Texas, United States of America; 4 Department of Pathology and Immunology, Baylor College of Medicine, Houston, Texas, United States of America; Consejo Nacional de Investigaciones Científicas y Técnicas: Consejo Nacional de Investigaciones Cientificas y Tecnicas, ARGENTINA

## Abstract

Phosphoinositide-dependent kinase-1 (PDK1) is a master kinase of the protein A, G, and C (AGC) family kinases that play important roles in regulating cancer cell proliferation, survival, and metabolism. Besides phosphorylating/activating AKT at the cell membrane in a PI3K-dependent manner, PDK1 also exhibits constitutive activity on many other AGC kinases for tumor-promoting activity. In the latter case, PDK1 protein levels dominate its activity. We previously reported that MAPK4, an atypical MAPK, can PI3K-independently promote AKT activation and tumor growth. Here, using triple-negative breast cancer (TNBC) cell models, we demonstrate that MAPK4 can also enhance PDK1 protein synthesis, thus phosphorylate/activate PDK1 substrates beyond AKT. This new MAPK4-PDK1 axis alone lacks vigorous tumor-promoting activity but cooperates with our previously reported MAPK4-AKT axis to promote tumor growth. Besides enhancing resistance to PI3K blockade, MAPK4 also promotes cancer cell resistance to the more stringent PI3K and PDK1 co-blockade, a recently proposed therapeutic strategy. Currently, there is no MAPK4 inhibitor to treat MAPK4-high cancers. Based on the concerted action of MAPK4-AKT and MAPK4-PDK1 axis in promoting cancer, we predict and confirm that co-targeting AKT and PDK1 effectively represses MAPK4-induced cancer cell growth, suggesting a potential therapeutic strategy to treat MAPK4-high cancers.

## Abstract 2

This study shows that besides activating AKT, MAPK4 promotes PDK1 protein synthesis and thus also activates PDK1 substrates beyond AKT to promote cancer. Co-targeting AKT and PDK1 can therefore repress MAPK4-induced cancer cell growth, suggesting a potential therapeutic strategy to treat MAPK4-high cancers, including a large subset of "triple negative" breast cancer.

## Introduction

The protein A, G, and C (AGC) family kinases consist of more than 60 evolutionarily related serine/threonine protein kinases. Many AGC kinases, such as phosphoinositide-dependent protein kinase-1 (PDK1), protein kinase B (AKT), serum and glucocorticoid-inducible kinases (SGK), protein kinase C (PKC), p70 ribosomal protein S6 kinase (S6K), and p90 ribosomal protein S6 kinase (RSK) play important roles in regulating cell proliferation, apoptosis, and metabolism [1]. Besides its activity in phosphorylating/activating AKT in the phosphatidylinositol 3-kinase (PI3K) pathway, PDK1 also exhibits constitutive activity in phosphorylating many other AGC kinases, such as S6K, SGK, PKC, and RSK for tumor-promoting activities [1,2]. In the latter case, PDK1 protein expression levels determine its activities. Therefore, it is important to understand the molecular mechanism regulating PDK1 protein levels, which remains elusive.

Mitogen-activated protein kinase 4 (MAPK4) is an atypical MAPK not well studied. We recently reported that MAPK4 can promote cancer by noncanonically activating AKT independent of the PI3K/PDK1 signaling axis [3]. The parallel actions of MAPK4 and PI3K-PDK1 in activating AKT predict MAPK4 activities in regulating cell response to PI3K blockade. Indeed, inhibiting MAPK4 sensitizes cancer cells to PI3K blockade [4]. However, it remains unknown why inhibiting MAPK4 (knockdown/knockout) greatly represses AKT phosphorylation/activation that the canonical PI3K pathway can also drive.

Triple-negative breast cancer (TNBC) is a devastating disease accounting for 15% to 20% of all breast cancer but with limited therapeutic options. We previously reported that MAPK4 is highly expressed in a large fraction of TNBC, and repressing MAPK4 is effective in inhibiting TNBC cell and xenograft growth [4]. Currently, there is no MAPK4-specific inhibitor(s) for potential clinical testing to treat TNBC. Further dissecting MAPK4 downstream signaling nodes, especially those with clinical inhibitor(s) being used in the clinic or tested in clinical trials, may provide an alternative route to treat MAPK4-high TNBC.

Here, using TNBC cell models, we report that besides directly phosphorylating/activating AKT (an MAPK4-AKT axis [3,4]), MAPK4 also greatly promotes PDK1 protein expression, representing an MAPK4-PDK1 axis to enhance PDK1 expression/activation. Blocking MAPK4 both inhibits the PI3K-independent MAPK4-AKT signaling axis [3,4] and represses PDK1 protein expression to block the canonical PI3K-PDK1-AKT pathway. Together, these lead to greatly repressed AKT phosphorylation/activation. We further demonstrate that by enhancing PDK1 protein expression/activity, MAPK4 also enhances PDK1-dependent but AKT-independent signaling. Accordingly, co-blockade of AKT and PDK1 largely blocks MAPK4 tumor-promoting activity. Our studies collectively identify a novel mechanism promoting PDK1 protein expression and further advance our knowledge of the molecular mechanism underlying the tumor-promoting activity of MAPK4, an emerging novel therapeutic target for human cancers.

## Results

### MAPK4 up-regulates PDK1 protein expression in cancer cells

**Fig 1:**
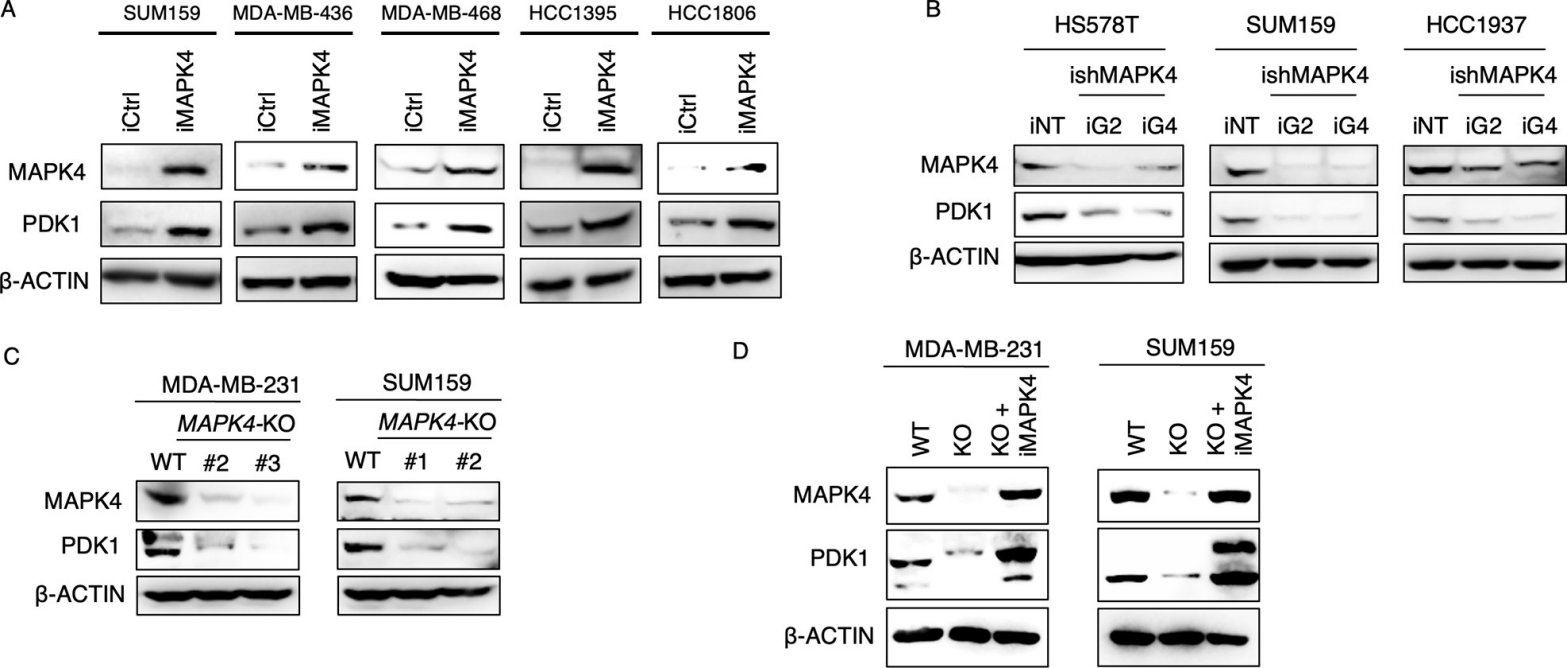
MAPK4 up-regulates PDK1 protein expression in cancer cells. Western blots on **(A)** engineered SUM159, MDA-MB-436, MDA-MB-468, HCC1395, and HCC1806 cells with 0.5 μg/ml Dox-induced ectopic expression of MAPK4 (iMAPK4) or control (iCtrl), (**B**) engineered HS578T, SUM159, and HCC1937 cells with 4 μg/ml Dox-induced knockdown of MAPK4 (iG2 and iG4) or control (iNT), (**C**) the parental vs. *MAPK4*-KO MDA-MB-231 cells (clone# 2, 3) and SUM159 cells (clone# 1, 2), and (**D**) the parental vs. *MAPK4*-KO MDA-MB-231 (clone# 3) and SUM159 cells (clone# 2, KO cells) vs. *MAPK4*-KO cells with rescued 0.5 μg/ml Dox-induced MAPK4 expression (KO+iMAPK4). All cells in Panel D were treated with 0.5 μg/ml Dox. Data are representative of at least 2–3 independent experiments. MAPK4, mitogen-activated protein kinase 4; PDK1, phosphoinositide-dependent kinase-1.

We have shown that MAPK4 can activate AKT independent of the PI3K pathway [3]. However, it stays unknown why inhibiting MAPK4 greatly represses AKT phosphorylation/activation that the canonical PI3K signaling can also drive. We have previously documented human TNBC cell lines with high, medium, or low MAPK4 expressions [4]. Overexpression of MAPK4 in the MAPK4-medium SUM159 and MAPK4-low MDA-MB-436, MDA-MB-468, HCC1395, and HCC1806 cells all enhanced PDK1 protein expression, suggesting an unexpected MAPK4-PDK1 signaling axis (Fig 1A). Accordingly, knockdown/knockout of MAPK4 in the MAPK4-medium SUM159 and MAPK4-high HS578T, HCC1937, and MDA-MB-231 cells reduced PDK1 protein expression (Fig 1B and 1C) and ectopic expression of MAPK4 in the *MAPK4*-KO MDA-MB-231 and SUM159 cells rescued PDK1 expression (Fig 1D). Neither overexpression nor knockdown of MAPK4 affected PDK1 mRNA expression in the examined cell lines (S1A Fig), suggesting that MAPK4 promotes PDK1 protein expression through a posttranscriptional process, such as regulation of protein synthesis and stability.

The molecular mechanism regulating PDK1 protein stability remains unknown until the recent identification of SPOP as an E3 ubiquitin ligase for PDK1 protein ubiquitination and degradation [5]. To examine whether MAPK4 plays a major role in regulating PDK1 protein stability, we first examined PDK1 protein stability in 7 TNBC cancer cell lines, including the MAPK4-low HCC1806, MDA-MB-468, HCC1395 cells, and the MAPK4-medium/high SUM159, MDA-MB-231, HCC1937, and HS578T cell lines. We observed that PDK1 protein is very stable in MDA-MB-231, HCC1937, HS578T, HCC1806, and MDA-MB-468 cells, while notable PDK1 protein degradation was only evident in SUM159 and HCC1395 cells after prolonged protein synthesis inhibitor cycloheximide (CHX) treatment (S1B Fig). As an additional control, we also confirmed a rapid degradation of MCL-1 and Cyclin D1 in the CHX-treated MDA-MB-231, HCC1937, HS578T, and SUM159 cells. Therefore, there appears a lack of correlation between MAPK4 protein expression levels and PDK1 protein stability. Besides, proteasome inhibitor MG132 treatment did not greatly affect PDK1 protein levels in either control or MAPK4-knockdown SUM159 and HS578T cells (S1C Fig). Together, our data suggest that MAPK4 neither regulates PDK1 mRNA expression nor notably affects PDK1 protein stability. Accordingly, we next examined whether MAPK4 enhances PDK1 protein translation.

### eIF4E mediates MAPK4 regulation of PDK1 protein expression

Most eukaryotic mRNAs carry a methyl-7-guanosine (m^7^G) cap at the 5′ end. The eukaryotic translation initiation complex eIF4F plays a critical role in protein synthesis from these m^7^G capped mRNAs. eIF4F translation initiation complex consists of eukaryotic translation initiation factor eIF4E, eIF4G, and eIF4A [6]. Among these factors, eIF4E is the least abundant initiation factor providing the rate-limiting step to bind to the m^7^G cap for translation initiation. Although m^7^G capped mRNA relies on eIF4E for translation, not all mRNAs are equally sensitive to altered eIF4E activity. There is a specific subset of “eIF4E-sensitive mRNAs” that often encode proteins involved in cell growth and survival [7]. Therefore, we investigated whether MAPK4-enhanced PDK1 protein expression is sensitive to altered eIF4E levels. Knockdown of eIF4E greatly repressed PDK1 protein expression in both the control and MAPK4-overexpressing HCC1806 and SUM159 cells (S2A Fig), indicating a critical role of eIF4E in PDK1 protein synthesis, including MAPK4-induced PDK1 protein expression.

eIF4E phosphorylation at Serine 209 (S209) may prime eIF4E activation to enhance the translation of certain mRNAs to promote cancer [8]. Hence, we next examined whether MAPK4 regulates eIF4E phosphorylation at S209. Dox-induced MAPK4 overexpression in SUM159, MDA-MB-468, HCC1395, and HCC1806 cells all greatly enhanced eIF4E S209 phosphorylation (S2B Fig). Conversely, knockdown of MAPK4 in HS578T, SUM159, and HCC1937 cells reduced such phosphorylation (S2C Fig). Furthermore, MAPK4 appeared to bind to eIF4E in the co-IP assays (S2D Fig) and GST-pulldown assays (S2E Fig). Together, these data suggest that MAPK4 can bind eIF4E and enhance its phosphorylation at S209.

### MNK1/2 inhibition blocks MAPK4-induced eIF4E S209 phosphorylation but does not affect MAPK4 enhancing PDK1 expression or cancer cell growth

MNK1 and MNK2 are the only known protein kinases catalyzing eIF4E phosphorylation at S209 [9–11]. MNK1/2-mediated eIF4E S209 phosphorylation is believed to be a key event promoting cancer, and MNK1/2-specific inhibitors such as eFT508 are being examined in cancer clinical trials [12]. Since we showed that MAPK4 overexpression enhances eIF4E phosphorylation at S209 and MAPK4 knockdown represses this phosphorylation (S2B and S2C Fig), a candidate working model would be that MAPK4 enhances PDK1 protein synthesis by promoting MNK1/2-mediated eIF4E S209 phosphorylation and activation. To test this hypothesis, we treated the cells with 2 different MNK1/2 inhibitors SLV-2436 (SEL201) and eFT508 [13,14]. Treatments using either inhibitor robustly blocked the basal and MAPK4-induced eIF4E S209 phosphorylation in HCC1806 and SUM159 cells, indicating that MNK1/2 are essential kinases for eIF4E S209 phosphorylation as reported and that MNK1/2 mediate MAPK4 activities in enhancing eIF4E S209 phosphorylation (S3A Fig). However, none of these inhibitor treatments affected basal or MAPK4-induced PDK1 protein levels in HCC1806 and SUM159 cells (S3A Fig). SLV-2436 and eFT508 treatments similarly repressed eIF4E S209 phosphorylation but exhibited little effect on PDK1 protein levels in wild type (WT) or *MAPK4*-KO SUM159 and MDA-MB-231 cells (S3B Fig). Finally, SLV-2436 and eFT508 treatments did not affect the basal or MAPK4-induced HCC1806 and SUM159 cell growth, nor the growth of WT or *MAPK4*-KO MDA-MB-231 cells (S3C and S3D Fig). Collectively, these data suggest that MAPK4 promotes MNK1/2-mediated eIF4E S209 phosphorylation; however, unexpectedly, eIF4E S209 phosphorylation does not play a notable role in MAPK4 promotion of PDK1 protein expression or tumor growth.

### PDK1 partially mediates MAPK4 tumor-promoting activity

**Fig 2:**
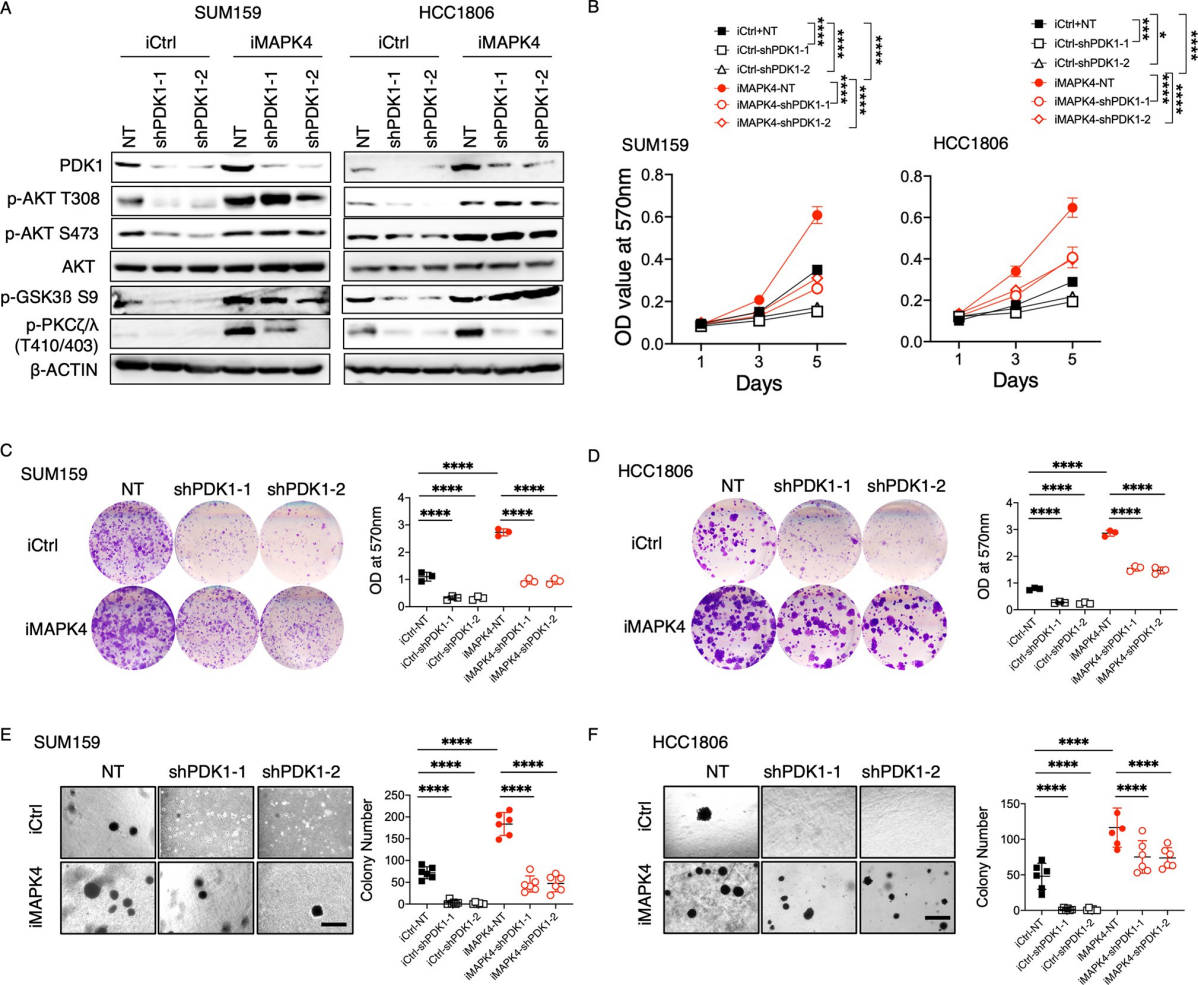
PDK1 partially mediates MAPK4 tumor-promoting activity. (**A**) Western blots, (**B**) proliferation assays, (**C, D**) plate clonogenic assays, and (**E, F**) soft-agar assays on the engineered SUM159 and HCC1806 cells with 0.5 μg/ml Dox-induced overexpression of MAPK4 (iMAPK4) or control (iCtrl). The cells were also engineered with stable knockdown of PDK1 (shPDK1-1, shPDK1-2) or control (NT). Quantification data as means ± SD. Scale bar: 500 μm. *P* values by two-way ANOVA followed by Sidak’s multiple comparisons. *P ≤ 0.05, ***P ≤ 0.001, ****P ≤ 0.0001. Data are representative of at least 3 independent experiments. The numerical values underlying the figures can be found in S1 Data. MAPK4, mitogen-activated protein kinase 4; PDK1, phosphoinositide-dependent kinase-1.

We have previously documented that MAPK4 can directly activate AKT independent of the canonical PI3K/PDK1 pathway to promote cancer growth [3]. Besides phosphorylating and activating AKT in a PI3K-dependent manner, PDK1 also exhibits constitutive activity on most substrates beyond AKT. Therefore, our newly discovered MAPK4-PDK1 signaling axis may exhibit PI3K-independent and/or AKT-independent tumor-promoting activities. Besides, by enhancing PDK1 protein expression, MAPK4 may further enhance the canonical PI3K-PDK1-AKT pathway to promote cancer growth. To investigate PDK1 function in mediating MAPK4 biology, we performed knockdown of PDK1 in control and MAPK4-overexpressing SUM159 and HCC1806 cells using 2 independent shRNAs. Both shRNAs produced robust knockdown of PDK1, which greatly inhibited the phosphorylation of PDK1 substrates PKCζ/λ in all these cells (Fig 2A). In contrast, knockdown of PDK1 only greatly reduced AKT phosphorylation in the control SUM159 and HCC1806 cells but had little effect in MAPK4-overexpressing cells, further supporting our previously reported MAPK4-AKT signaling axis independent of PI3K/PDK1 [3]. In accord with this, while knockdown of PDK1 profoundly repressed control SUM159 and HCC1806 cell growth, it only partially inhibited MAPK4-induced cell growth (Fig 2B–2F). We conclude that by blocking both the PI3K/PDK1-induced AKT phosphorylation/activation and the PI3K-independent and/or AKT-independent PDK1 signaling cascade, knockdown of PDK1 is very effective in inhibiting control cell growth. In contrast, knockdown of PDK1 leaves the MAPK4-AKT signaling axis largely intact in the MAPK4-overexpressing cells, which may account for the partially maintained tumor cell growth. Finally, these data also support that PDK1 partially mediates MAPK4 tumor-promoting activity.

### Overexpression of PDK1 partly rescues *MAPK4*-KO tumor cell growth and reduces their sensitivity to PI3K blockade

**Fig 3:**
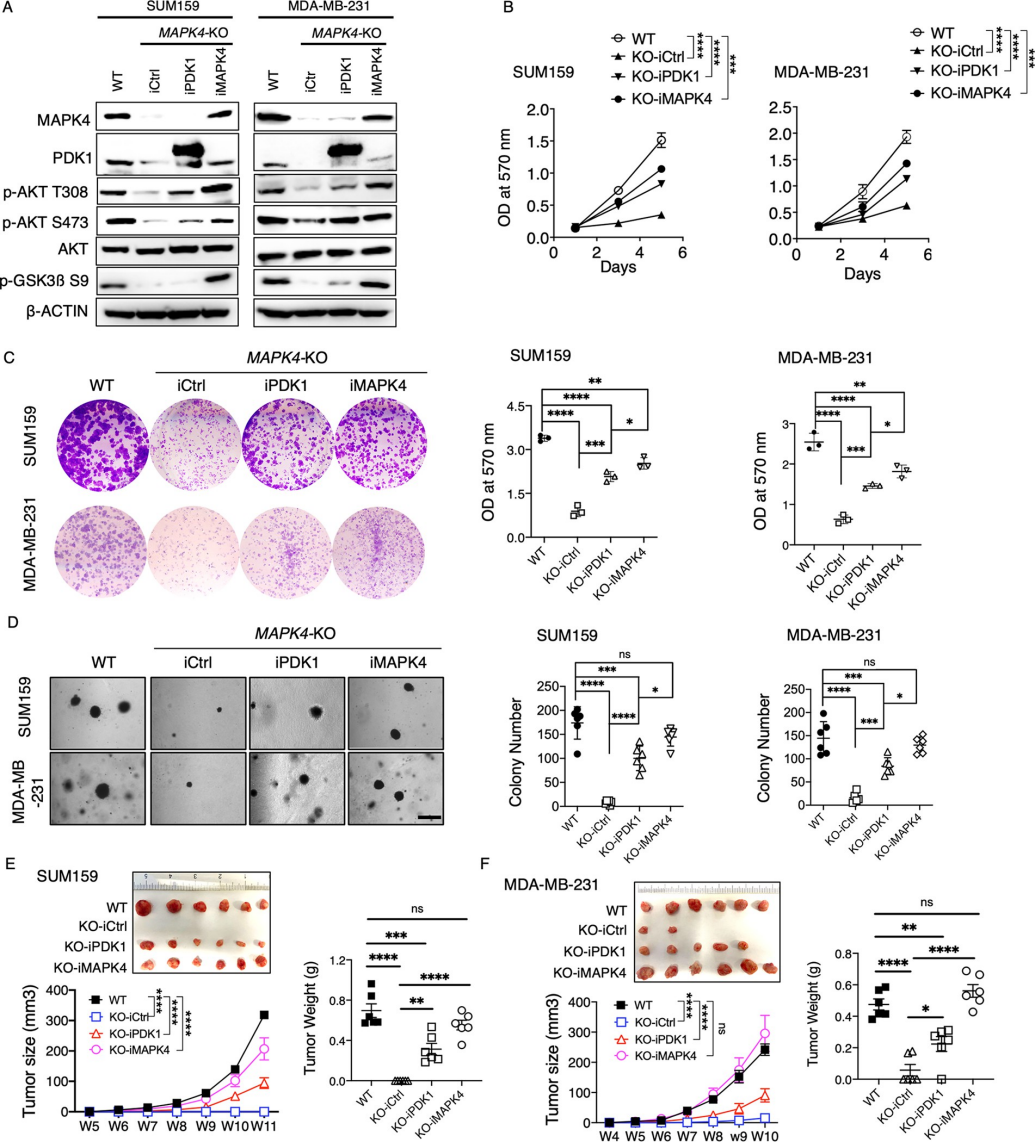
Overexpression of PDK1 partly rescues *MAPK4*-KO tumor cell growth. (**A**) Western blots, (**B**) proliferation assays, (**C**) plate colony formation assays, and (**D**) soft-agar assays on WT or engineered *MAPK4*-KO SUM159 and MDA-MB-231 cells with 0.5 μg/ml Dox-induced expression of PDK1 (iPDK1), MAPK4 (iMAPK4), or control (iCtrl). Scale bar: 500 μm. (**E**, **F**) 1 × 10^6^ WT or the engineered *MAPK4*-KO SUM159 and MDA-MB-231 cells with Dox-inducible expression of PDK1 (iPDK1), MAPK4 (iMAPK4), or control (iCtrl) in 1:2 Matrigel were injected into the mammary fat pad of SCID mice. All mice also received 0.5 mg/ml Dox in drinking water. Tumors were measured and harvested as indicated. Shown are the tumors’ images at collection, growth curve (means ± SEM), and weights. Quantification data as means ± SD other than otherwise indicated. *P* values by one-way ANOVA followed by Sidak’s multiple comparisons. *P ≤ 0.05, **P ≤ 0.01, ***P ≤ 0.001, ****P ≤ 0.0001. ns, not significant. Data are representative of at least 3 independent experiments. The numerical values underlying the figures can be found in S1 Data. MAPK4, mitogen-activated protein kinase 4; PDK1, phosphoinositide-dependent kinase-1; WT, wild type.

To further evaluate PDK1’s function in mediating MAPK4 tumor-promoting activity, we overexpressed PDK1 in the *MAPK4*-KO SUM159 and MDA-MB-231 cells. As a positive control, we also ectopically expressed MAPK4 in these *MAPK4*-KO cells to levels comparable to WT cells (Fig 3A). As expected, ectopic MAPK4 expression largely rescued PDK1 protein expression and AKT phosphorylation in the *MAPK4*-KO cells (Fig 3A). In contrast, PDK1 overexpression at levels considerably higher than WT cells could only partially rescue AKT phosphorylation (Fig 3A). In accord with these, overexpression of PDK1 could only partially rescue the growth, including anchorage-independent growth of the *MAPK4*-KO SUM159 and MDA-MB-231 cells in vitro and xenograft growth in vivo (Fig 3B–3F). Finally, overexpression of PDK1 appeared less potent than MAPK4 in rescuing the growth of these *MAPK4*-KO cells/xenografts (Fig 3B–3F), which are consistent with the notion that PDK1 only partially mediates MAPK4 tumor-promoting activity.

**Fig 4:**
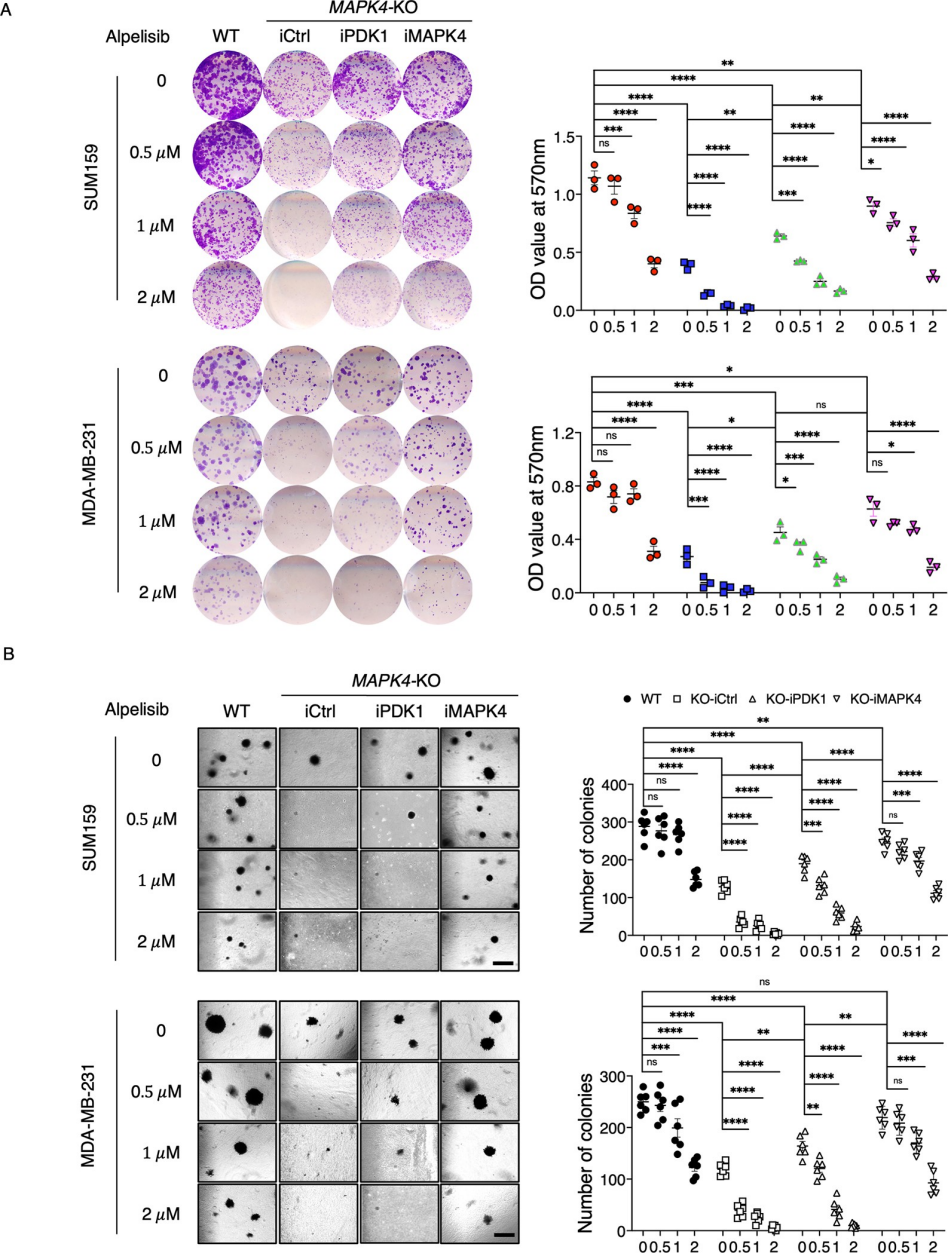
Overexpression of PDK1 partly rescues *MAPK4*-KO tumor cell resistance to PI3K blockade. (**A**) Plate colony formation assays and (**B**) soft-agar assays on WT or engineered *MAPK4*-KO SUM159 and MDA-MB-231 cells with 0.5 μg/ml Dox-induced expression of PDK1 (iPDK1), MAPK4 (iMAPK4), or control (iCtrl). Scale bar: 500 μm. Quantification data as means ± SD. *P* values by two-way ANOVA followed by Sidak’s multiple comparisons. *P ≤ 0.05, **P ≤ 0.01, ***P ≤ 0.001, ****P ≤ 0.0001. ns, not significant. Data are representative of at least 3 independent experiments. The numerical values underlying the figures can be found in S1 Data. MAPK4, mitogen-activated protein kinase 4; PDK1, phosphoinositide-dependent kinase-1; WT, wild type.

We have previously demonstrated that overexpression of MAPK4 enhances cancer cell resistance to PI3K blockade [4]. We next examined whether overexpression of PDK1 reduces *MAPK4*-KO cell sensitivity to PI3K inhibition. As we previously reported, compared to parental cells, the *MAPK4*-KO SUM159 and MDA-MB-231 cells grew slower and were much more sensitive to PI3K inhibitor Alpelisib treatment (Fig 4A and 4B). Rescued MAPK4 expression in these *MAPK4*-KO cells largely restored their growth and resistance to Alpelisib. In contrast, overexpression of PDK1 only partially restored their growth and resistance to Alpelisib (Fig 4A and 4B). Together, these data suggest that PDK1 may only partially mediate MAPK4 activities in promoting tumor cell growth and resistance to PI3K blockade.

### MAPK4-induced PDK1 protein expression alone lacks robust activity but cooperates with AKT to promote tumor cell growth

**Fig 5:**
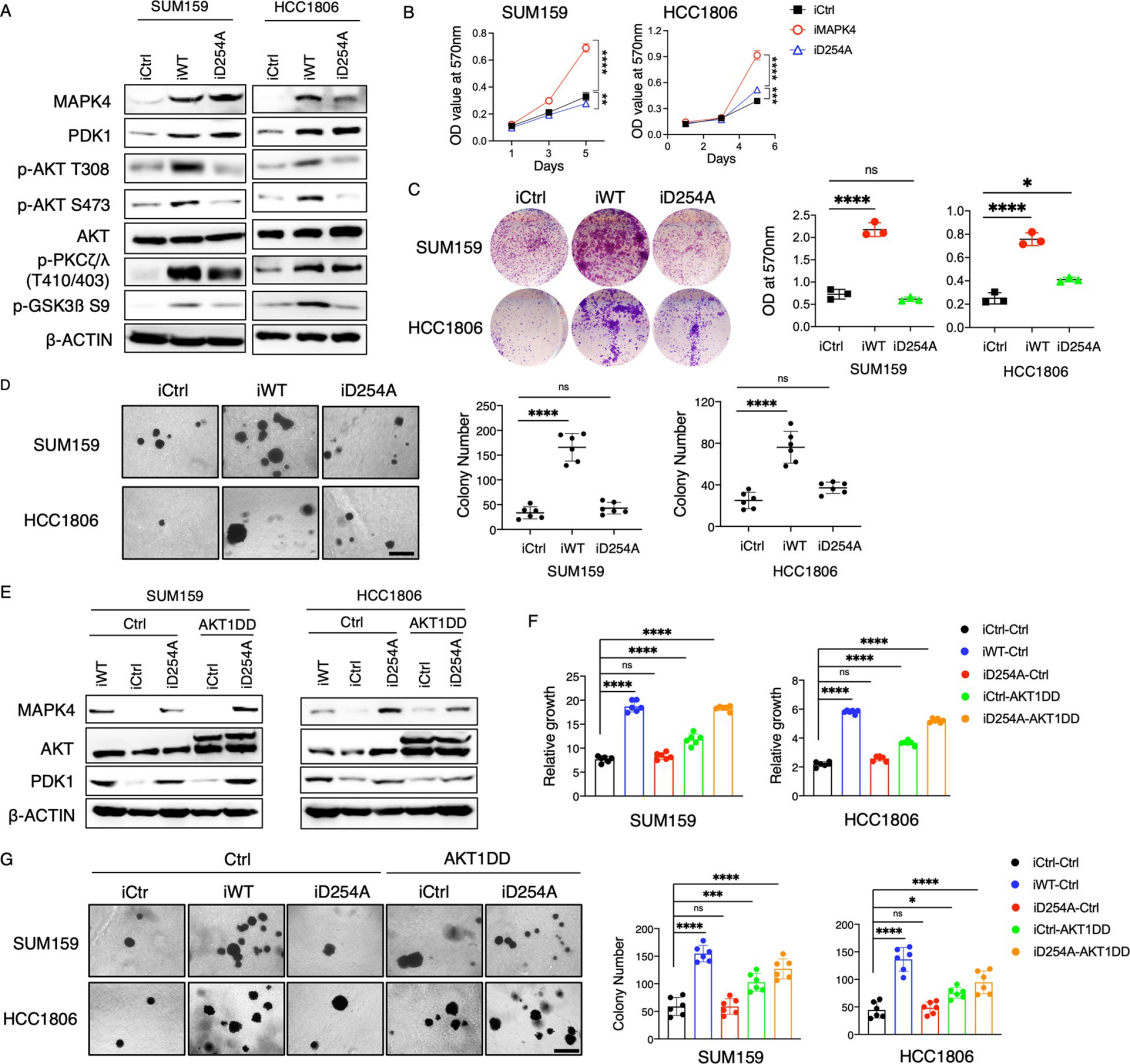
MAPK4-induced PDK1 works with AKT to promote tumor cell growth. (**A**) Western blots, (**B**) proliferation assays, (**C**) plate colony formation assays, and (**D**) soft-agar assays on engineered SUM159 and HCC1806 cells with 0.5 μg/ml Dox-induced overexpression of MAPK4 (iWT), MAPK4^D254A^ (iD254A), or control (iCtrl). Scale bar: 500 μm. (**E**) Western blots, (**F**) proliferation assays, and (**G**) soft-agar assays on engineered SUM159 and HCC1806 cells with 0.5 μg/ml Dox-induced overexpression of MAPK4 (iWT), MAPK4^D254A^ (iD254A), or control (iCtrl). These cells were also infected with lentivirus expressing AKT1-DD mutant or control. Quantification data as means ± SD. *P* values by one-way ANOVA followed by Sidak’s multiple comparisons. *P ≤ 0.05, **P ≤ 0.01, ***P ≤ 0.001, ****P ≤ 0.0001. ns, not significant. Data are representative of at least 3 independent experiments. The numerical values underlying the figures can be found in S1 Data. MAPK4, mitogen-activated protein kinase 4; PDK1, phosphoinositide-dependent kinase-1.

In our rescue studies (Figs 3 and 4), we overexpressed PDK1 in *MAPK4*-KO SUM159 and MDA-MB-231 cells to levels considerably higher than those of the WT cells. Yet, we can only partially rescue the MAPK4-mediated biology, such as promoting cancer cell/xenograft growth and their resistance to PI3K blockade. To critically define whether MAPK4-induced PDK1 at physiologically relevant levels regulates cancer cell growth and their response to PI3K blockade, we also used our previously described MAPK4^D254A^ mutant lacking affinity to AKT to address this question [3]. When ectopically expressed in SUM159 and HCC1806 cells, MAPK4^D254A^ exhibited similar activities as WT MAPK4 in promoting PDK1 protein expression and activation (phosphorylation of PKCζ/λ, Fig 5A). However, consistent with our previous observations, MAPK4^D254A^ largely lost its activities in enhancing AKT phosphorylation and promoting SUM159 and HCC1806 cell growth, including anchorage-independent growth (Fig 5A–5D). These data suggest that without MAPK4 directly engaging/activating AKT (MAPK4-AKT axis), MAPK4-induced PDK1 protein expression/activation (MAPK4-PDK1 axis) is not sufficient to promote robust cancer cell growth.

To further define whether MAPK4-induced PDK1 (MAPK4-PDK1 axis) plays any remarkable roles in promoting cancer cell growth, especially when combined with AKT activation, we investigated how co-expression of MAPK4^D254A^ (with activation of MAPK4-PDK1 axis) and a constitutively activated AKT1 (AKT1^T308D/S473D^, AKT1-DD, to simulate MAPK4-AKT axis) affects SUM159 and HCC1806 cell growth. While ectopic expression of AKT1-DD alone showed some activity, co-expression of MAPK4^D254A^ further enhanced cell growth, and the activity of co-expressed MAPK4^D254A^ and AKT1-DD largely recapitulated WT MAPK4 tumor-promoting activity in the proliferation assays (Fig 5E and 5F). Together, these data suggest that while MAPK4-induced PDK1 protein expression (MAPK4-PDK1 axis) alone is not sufficient to robustly promote AKT phosphorylation/activation and tumor cell growth, it does provide a route to enhance the tumor-promoting activity of activated AKT. Lastly, co-expression of MAPK4^D254A^ and AKT1-DD only partially recapitulated the tumor-promoting activity of WT MAPK4 in the soft-agar assays, suggesting that MAPK4 activation of additional signaling cascade beyond PDK1 and AKT may be important to promote tumor cell anchorage-independent growth (Fig 5G).

### MAPK4 promotes cancer cell resistance to combined PI3K and PDK1 blockade

Castel and colleagues reported that inhibition of PDK1 sensitizes cancer cells to PI3K blockade [15]. Therefore, a co-blockade of PI3K and PDK1 may provide a more effective therapeutic approach to treat cancers. Since MAPK4 can PI3K/PDK1-independently activate AKT, we predicted and experimentally validated that MAPK4 renders cancer cell resistance to PI3K blockade [4]. In contrast, how MAPK4 affects cancer cell response to the more stringent co-blockade of PI3K and PDK1 remains unknown.

**Fig 6:**
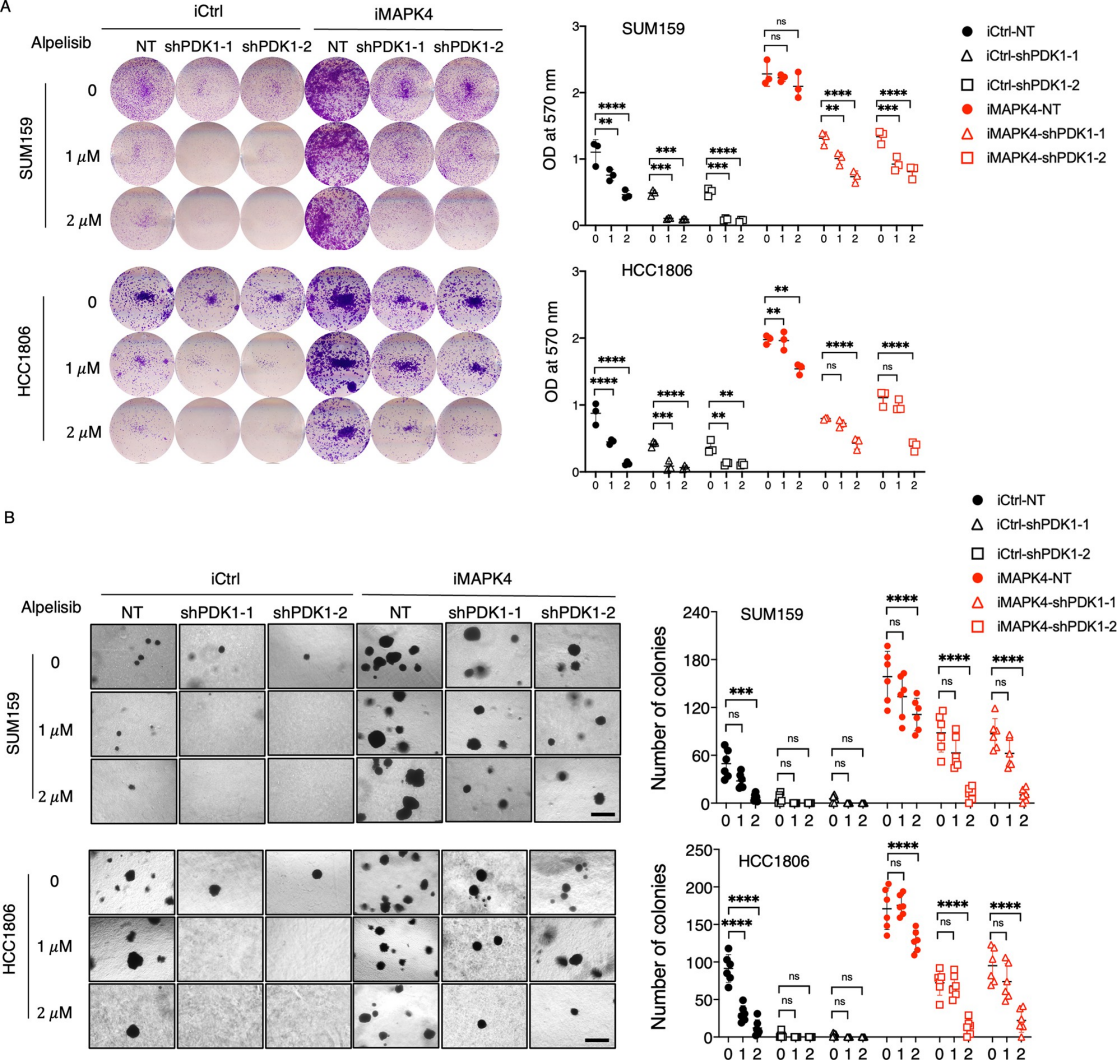
Knockdown of PDK1 partially sensitizes MAPK4-overexpressing tumor cells to PI3K blockade. (**A**) Plate colony formation assays and (**B**) soft-agar assays on the engineered SUM159 and HCC1806 cells with 0.5 μg/ml Dox-induced overexpression of MAPK4 (iMAPK4) or control (iCtrl), also with stable knockdown of PDK1 (shPDK1-1 and shPDK1-2) or control (NT). The cells were also treated with PI3K inhibitor Alpelisib (1 μm or 2 μm) or DMSO control. Scale bar: 500 μm. Quantification data as means ± SD. *P* values by two-way ANOVA followed by Sidak’s multiple comparisons. **P ≤ 0.01, ***P ≤ 0.001, ****P ≤ 0.0001. ns, not significant. Data are representative of at least 3 independent experiments. The numerical values underlying the figures can be found in S1 Data. MAPK4, mitogen-activated protein kinase 4; PDK1, phosphoinositide-dependent kinase-1.

To critically address this question, we first examined how PDK1 knockdown affects control and MAPK4-overexpressing SUM159 and HCC1806 cell response to PI3K inhibitor Alpelisib. In agreement with our previous studies, overexpression of MAPK4 both greatly enhanced SUM159 and HCC1806 cell growth and rendered them resistant to PI3K blockade (Fig 6A and 6B). Knockdown of PDK1 in control cells both reduced their growth and greatly sensitized them to Alpelisib treatments, as expected [15]. Knockdown of PDK1 also reduced the growth of MAPK4-overexpressing cells, as we described in Fig 2. In contrast, the PDK1-knockdown MAPK4-overexpressing cells largely maintained their growth in the presence of 1 μm Alpelisib, thus, were resistant to 1 μm Alpelisib treatment (Fig 6A and 6B). These cells were also partially resistant to 2 μm Alpelisib treatments (Fig 6A and 6B). For example, while both 1 and 2 μm Alpelisib treatments essentially wiped off the anchorage-independent growth of PDK1-knockdown control SUM159 and HCC1806 cells, the PDK1-knockdown MAPK4-overexpressing cells maintained robust growth when treated with 1 μm Alpelisib and produced numerous clones when treated with 2 μm Alpelisib (Fig 6B). Collectively, these data suggest that MAPK4 both PDK1-independently and -dependently regulates tumor cell response to PI3K blockade, and MAPK4 overexpression renders cancer cells at least partially resistant to PI3K and PDK1 co-blockade.

**Fig 7:**
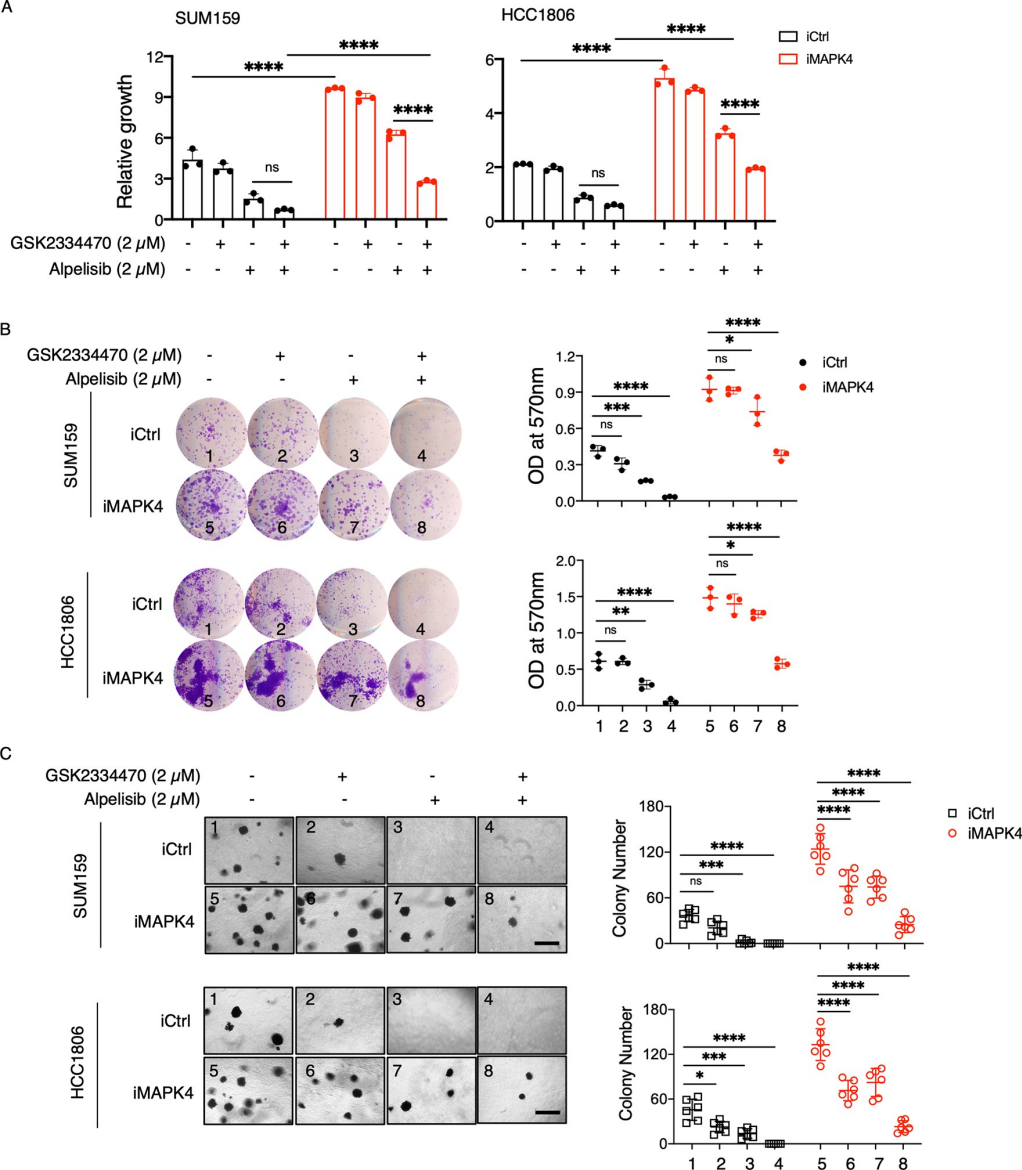
MAPK4 promotes cancer cell resistance to PI3K and PDK1 co-blockade. (**A**) Proliferation assays, (**B**) plate colony formation assays, and (**C**) soft-agar assays on the engineered SUM159 and HCC1806 cells with 0.5 μg/ml Dox-induced overexpression of MAPK4 (iMAPK4) or control (iCtrl). The cells were also treated with DMSO control, PDK1 inhibitor GSK2334470 (2 μm), PI3K inhibitor Alpelisib (2 μm), or both inhibitors. Scale bar: 500 μm. Quantification data as means ± SD. *P* values by two-way ANOVA followed by Sidak’s multiple comparisons. *P ≤ 0.05, **P ≤ 0.01, ***P ≤ 0.001, ****P ≤ 0.0001. ns, not significant. Data are representative of at least 3 independent experiments. The numerical values underlying the figures can be found in S1 Data. MAPK4, mitogen-activated protein kinase 4; PDK1, phosphoinositide-dependent kinase-1.

To confirm our above observations further, we also treated MAPK4-overexpressing and control SUM159 and HCC1806 cells with PI3K inhibitor Alpelisib (2 μm), PDK1 inhibitor GSK2334470 (2 μm), and in combination. As predicted [15], co-treatment showed remarkable activity in repressing control tumor cell growth, resulting in few growth in the plate clonogenic assays and soft-agar assays (Fig 7A–7C). In contrast, MAPK4 overexpression both promoted cell growth and rendered tumor cells at least partially resistant to Alpelisib and GSK2334470 co-treatment (Fig 7A–7C). These data further support MAPK4 roles in enhancing both tumor cell growth and at least partial resistance to combined PI3K and PDK1 blockade. Accordingly, compared with WT cells, the *MAPK4*-KO SUM159 cells were more sensitive to PI3K inhibitor Alpelisib (1 μm), PDK1 inhibitor GSK2334470 (2 μm), and in combination, and ectopic expression of MAPK4 in these *MAPK4*-KO cells rescued their growth to WT levels (S4 Fig).

### Co-targeting AKT and PDK1 is effective in repressing MAPK4-induced cancer cell growth

**Fig 8:**
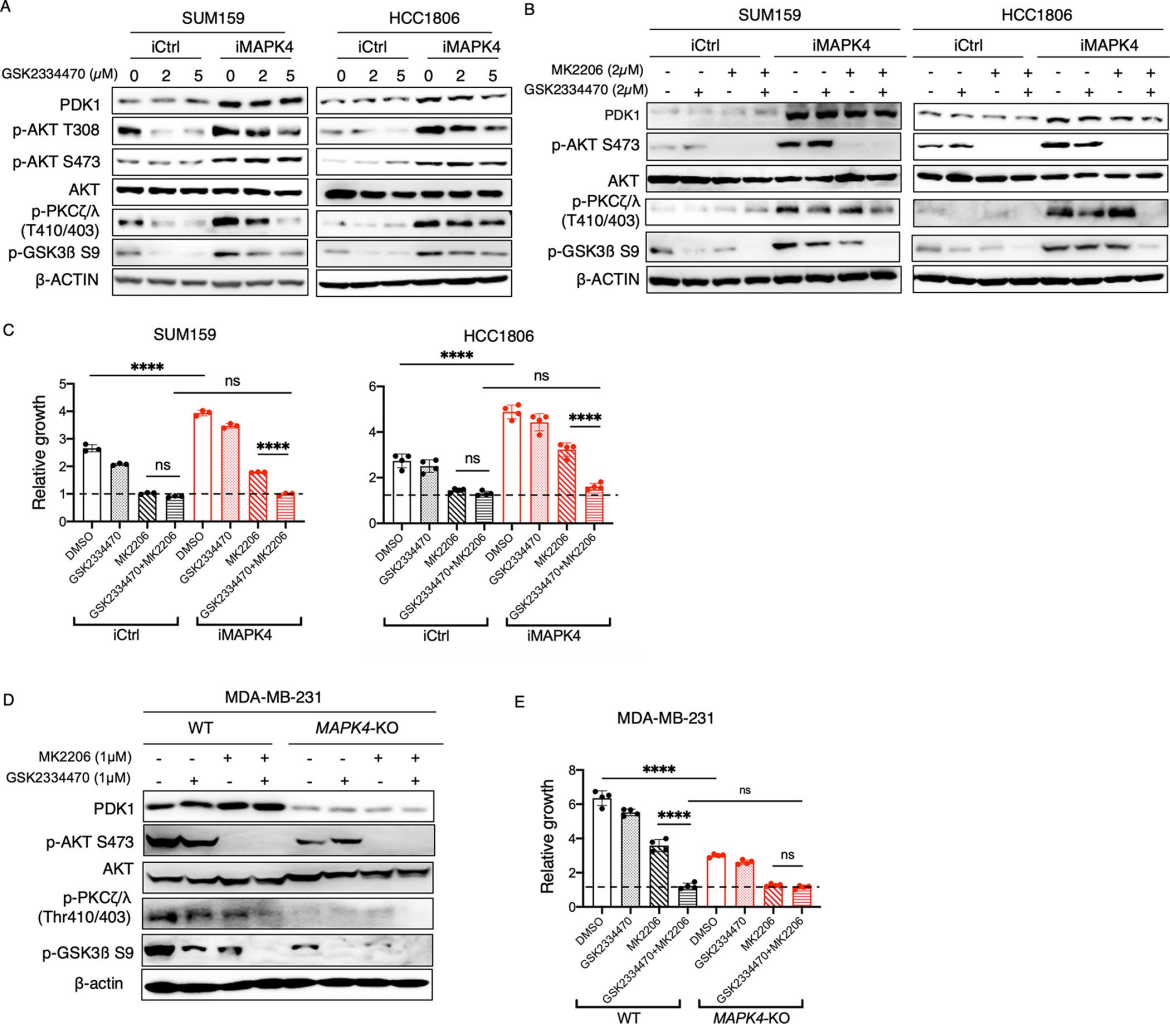
Co-targeting AKT and PDK1 is effective in repressing MAPK4-induced cancer cell growth. (**A**) Western blot on the engineered SUM159 and HCC1806 cells with 0.5 μg/ml Dox-induced overexpression of MAPK4 (iMAPK4) or control (iCtrl), treated with 2 μm or 5 μm of PDK1 inhibitor GSK2334470 or DMSO control. (**B**) Western blots and (**C**) proliferation assays on the engineered SUM159 and HCC1806 cells with 0.5 μg/ml Dox-induced overexpression of MAPK4 (iMAPK4) or control (iCtrl). The cells were also treated with DMSO control, PDK1 inhibitor GSK2334470 (2 μm), AKT inhibitor MK2206 (2 μm), or both inhibitors. (**D**) Western blots and **(E)** proliferation assays on the WT and *MAPK4*-KO MDA-MB-231 cells treated with DMSO control, PDK1 inhibitor GSK2334470 (1 μm), AKT inhibitor MK2206 (1 μm), or both inhibitors. Data are means ± SD from at least 3 separate experiments. *P* values by two-way ANOVA followed by Sidak’s multiple comparisons. ns, not significant, ****P < 0.0001. The numerical values underlying the figures can be found in S1 Data. MAPK4, mitogen-activated protein kinase 4; PDK1, phosphoinositide-dependent kinase-1; WT, wild type.

We have previously demonstrated that AKT activation is essential for supporting MAPK4 tumor-promoting activity [3]. Although high concentration of AKT inhibitor treatment is very effective in inhibiting both the control and MAPK4-overexpressing cancer cell growth, the potential toxicity of AKT inhibitors at high concentrations is predicted to limit their therapeutic potential. Since our newly identified MAPK4-PDK1 axis can work with the MAPK4-AKT axis to promote cancer growth, we examined whether co-targeting PDK1 and AKT using PDK1 inhibitor GSK2334470 and AKT inhibitor MK2206 will be a valid approach to block MAPK4 tumor-promoting activities. We first confirmed that PDK1 inhibitor GSK2334470 (2 μm and 5 μm) can inhibit MAPK4-induced PDK1 activation (PDK1-mediated PKCζ/λ phosphorylation) in SUM159 and HCC1806 cells (Fig 8A). We then tested the effects of GSK2334470 at a suboptimal concentration (2 μm) and AKT inhibitor MK2206 (2 μm), alone or in combination, on MAPK4-regulated signaling cascade and SUM159 and HCC1806 cell growth. The MK2206 (2 μm) treatment potently blocked both the basal and MAPK4-induced AKT phosphorylation, greatly repressed control cell growth but only partially blocked MAPK4-induced cell growth (Fig 8B and 8C). As expected, the suboptimal GSK2334470 (2 μm) treatment reduced PKCζ/λ phosphorylation with minor effects on cell growth. In contrast, the combined MK2206 and GSK2334470 treatment produced robust inhibition of both basal and MAPK4-induced PKCζ/λ and GSK3β phosphorylation and blocked MAPK4-induced SUM159 and HCC1806 cell growth (Fig 8B and 8C). We observed a similar robust activity of PDK1 inhbitor GSK2334470 (1 μm) and AKT inhibitor MK2206 (1 μm) co-treatment in blocking the MAPK4-high MDA-MB-231 cell growth. In contrast, the *MAPK4-KO* MDA-MB-231 cells were already sensitive to MK2206 treatment (Fig 8D and 8E).

## Discussion

Although PDK1 needs to bind PIP3 and activate AKT at the cell membrane in the PI3K-PDK1-AKT signaling pathway, PDK1 is constitutively activated, and its protein levels dominate its activity on most targets beyond AKT. Therefore, it is crucial to understand how PDK1 protein expression is regulated in human cancers. Here, using human TNBC cell line models, we uncovered an unexpected robust pathway for MAPK4 promoting PDK1 protein expression. We also made similar observations on MAPK4 enhancing PDK1 protein levels in human non-small cell lung cancer H1299 and H157 cells, colorectal cancer HCT116 cells, and prostate cancer DU145 cells (S5 Fig), suggesting the MAPK4-PDK1 axis beyond TNBC. By enhancing PDK1 protein expression, MAPK4 both activates PDK1 substrates beyond AKT and supports the PI3K-PDK1-AKT signaling cascade to regulate cancer cell biology.

We have shown that MAPK4 can activate AKT independent of the PI3K pathway [3]. However, it was unknown why inhibiting MAPK4 (knockdown/knockout of MAPK4) robustly represses AKT phosphorylation/activation that the canonical PI3K pathway can also drive. Our currently reported MAPK4-PDK1 signaling axis may provide an answer to that question. For example, knockdown/knockout of MAPK4 can directly repress AKT phosphorylation/activation by blocking the MAPK4-AKT axis. Besides, by blocking the MAPK4-PDK1 axis, knockdown/knockout of MAPK4 also decreases PDK1 protein expression to inhibit the canonical PI3K-PDK1-AKT pathway, further reducing AKT phosphorylation/activation.

Previous reports have well documented the AKT-independent tumor-promoting activities of PDK1. For example, the PDK1-PLK1-MYC, PDK1-SGK1, and PDK1-PKCα signaling axis have been shown to promote tumor growth, therapy resistance, and/or confer oncogenic transformation [15–17]. Therefore, theoretically, by enhancing PDK1 protein expression, the MAPK4-PDK1 signaling axis will promote cancer by activating the above PDK1-dependent but AKT-independent tumor-promoting pathway. However, the MAPK4^D254A^ mutant, which can promote PDK1 protein expression but cannot directly bind and activate AKT, exhibited limited tumor-promoting activity (Fig 5). These data suggest that the AKT-independent MAPK4-PDK1 signaling axis alone is not sufficient for robust tumor-promoting activity.

MNK1 and MNK2 are the only known kinases that can phosphorylate eIF4E at S209, which has been shown crucial to promote the synthesis of certain proteins important in cell growth [8]. Therefore, several MNK1/2 inhibitors are being tested in clinical trials [12]. We find that MAPK4 promotes eIF4E S209 phosphorylation, and MNK1/2 inhibitor treatments (SLV-2436 and eFT508) robustly block MAPK4-induced eIF4E phosphorylation at S209. These suggest a potential MAPK4-MNK1/2-eIF4E signaling axis critical for MAPK4 promoting eIF4E S209 phosphorylation. Interestingly, neither of the 2 MNK1/2 inhibitors tested (SLV-2436 and eFT508) exhibited notable activity in inhibiting MAPK4-induced PDK1 protein expression or tumor cell growth (S3 Fig). These indicate that MNK1/2-mediated eIF4E S209 phosphorylation is not required for MAPK4 activities in promoting PDK1 protein expression or cell growth. In contrast, knockdown of eIF4E repressed MAPK4-induced PDK1 protein expression, suggesting a functional MAPK4-eIF4E-PDK1 axis that remains to be fully characterized (S2A Fig). Finally, despite robust activities in blocking eIF4E phosphorylation at S209, none of the 2 MNK1/2 inhibitors exhibited notable tumor-inhibiting activities in any of the tumor cell lines we tested, including the MAPK4-low and *MAPK4*-KO cancer cell lines (S3C and S3D Fig). Our current data at least raise caution for clinical trials testing MNK1/2 inhibitors in treating cancers without some type(s) of stratification.

Our previously reported PI3K-independent MAPK4-AKT signaling axis provides a direct mechanism for MAPK4 driving tumor cell resistance to PI3K blockade [4]. It has been reported that combined PI3K and PDK1 blockade is highly effective in blocking cancer cell growth, which may provide an improved therapeutic approach to treat cancers [15]. We predict that the MAPK4-AKT axis may also sustain AKT phosphorylation/activation and provide MAPK4-high cancer cells resistance to combined PI3K and PDK1 inhibitors treatment. Indeed, our data confirm this, suggesting the necessity of considering MAPK4 expression status in future clinical trials testing PI3K and PDK1 co-blockade.

PDK1 clearly partially mediates MAPK4 tumor-promoting activity (Fig 2). However, the MAPK4-PDK1 axis itself (expression of MAPK4^D254A^) lacks robust activity in phosphorylating/activating AKT and driving tumor cell growth. When combined with AKT activation (expression of a constitutively activated AKT-DD), MAPK4^D254A^ exhibits strong tumor-promoting activity (Fig 5). These data suggest that a concerted action of MAPK4-AKT and MAPK4-PDK1 signaling axis underlies MAPK4 overall tumor-promoting activity. We also noticed that while dual pharmacological blockade of AKT and PDK1 is sufficient to almost completely block the growth of MAPK4-overexpressing cells (Fig 8C), dual genetic activation of AKT and PDK1 (overexpression of MAPK4^D254A^ and PDK1, Fig 5F and 5G) does not fully recapitulate MAPK4 overexpression. These suggest that MAPK4 activates additional signaling cascade(s) beyond PDK1 and AKT for full tumor-promoting activity. In this case, dual activation of AKT and PDK1 is necessary (Fig 8C) but not sufficient (Fig 5F and 5G) for the full tumor-promoting activity of MAPK4.

We have previously reported that inhibiting MAPK4 (knockdown or knockout) is very effective in suppressing the growth of various MAPK4-high cancer cells, including breast cancer, prostate cancer, non-small cell lung cancer, and colon cancer [3,4,18]. Specifically, MAPK4 is highly expressed in a large fraction of TNBC, which accounts for 15% to 20% of all breast cancer but with limited therapeutic options [4]. Currently, there is no MAPK4-specific inhibitor(s), which prevents us from testing our discoveries in the clinic. In contrast, many AKT and PDK1 inhibitors are being tested in cancer clinical trials. Based on the concerted actions of MAPK4-AKT and MAPK4-PDK1 signaling axis in driving MAPK4 tumor-promoting activities, we tested and confirmed that co-blockade of AKT and PDK1 effectively represses MAPK4-induced cancer cell growth (Fig 8). This supports an exciting therapeutic opportunity to use AKT and PDK1 inhibitors to treat MAPK4-high cancers, such as a large fraction of TNBC.

## Materials and methods

### Ethics statement

All animal studies were approved by the Institutional Animal Care and Use Committee of Baylor College of Medicine under protocol AN-5220.

### Reagents and antibodies

The antibodies against p-AKT T308 (Catalog 13038), p-AKT S473 (Catalog 4060), AKT (Catalog 9272), PDK1 (Catalog 3062S), eIF4E (catalog 2067), p-eIF4E (Catalog 9741), p-PKCζ/λ (Thr410/403) (Catalog 9378), GSK3β (Catalog 9315), p-GSK3β S9 (Catalog 9336), MCL-1 (Catalog 5453), and Cyclin D1 (Catalog 2978) were from Cell Signaling Technology. Other antibodies used include anti-MAPK4 (Abgent, Catalog AP7298b), anti-MAPK4 (Origene, Catalog TA505872), anti-PDK1 (Santa Cruz, Catalog sc-17765), anti-HA (Santa Cruz, Catalog sc-805), anti-FLAG (Millipore Sigma, catalog F3165), and anti-β-ACTIN (Abclonal, Catalog AC026). The kinase inhibitors used include PI3K inhibitor Alpelisib/BYL-719 (MedChemExpress, Catalog HY-15244), PDK1 inhibitor GSK2334470 (MedChemExpress, Catalog HY-14981), AKT inhibitor MK2206 (Selleckchem, Catalog S1078), and MNK1/2 inhibitors SLV-2436/SEL201 (Medchemexpres, Catalog HY-112113) and EFT-508 (Selleckchem, Catalog S8275). EZview Red Anti-FLAG M2 Affinity Gel (catalog F2426) and MG132 (catalog M7449) were purchased from Millipore Sigma.

### Plasmids

The pInducer20-YF vector was constructed as previously described [3]. pRK5 vector was provided by Dr. Xin-Hua Feng at Baylor College of Medicine, Houston, Texas. The CDS of PDK1 or eIF4E was PCR amplified from the SUM159 cell cDNA, cloned into the pInducer20-YF vector between the MluI and SalI sites, then sequencing verified. The pGIPZ lentiviral shRNA constructs for knockdown of human PDK1 were purchased from Open Biosystem (Thermo Fisher Scientific).

### Cell culture, transfection, lentivirus infection

The human TNBC SUM159, MDA-MB-231, HCC1937, HCC1806, HCC1395, MDA-MB-468, HS578T cells, non-small cell lung cancer H1299, H157 cells, colorectal cancer HCT116 cells, and prostate cancer DU145 cells were acquired from ATCC. MDA-MB-231, HCC1937, MDA-MB-468, HS578T, and DU145 cells were cultured in DMEM supplemented with 10% FBS. HCC1806, HCC1395, H1299, H157, and HCT116 cells were cultured in RPMI1640 supplemented with 10% FBS (Hyclone or Invitrogen). SUM159 cells were cultured in F12 supplemented with 10% FBS. The *MAPK4*-knockout (KO) SUM159, MDA-MB-231, H1299, and H157 cell lines were described previously [3,4]. The pCDH based lentiviral constructs were used for lentivirus-mediated stable overexpression of MAPK4 in the *MAPK4-*KO cell as described before [3,4].

LipoD293 DNA transfection reagent (SignaGen Laboratories, catalog SL100668) was used for DNA transfection. For lentiviral mediated gene delivery, the packaging mix of lentiviral-based expression vectors pMD2.G and psPAX2 were transfected into the HEK293T cells. Harvested at 48 or 96 h after the transfection, viruses in the conditioned media were collected and then filtered (0.45 μm). Before drug selection, the targeted cells were infected with the viral media for 3 days, then expanded for further assays. Stable and doxycycline (Dox)-inducible overexpression of MAPK4, PDK1, or eIF4E cells were established using the pCDH and pInducer20-YF-based lentiviral gene delivery systems. Lentiviral-mediated stable or Dox-inducible knockdown of MAPK4, PDK1, or eIF4E cells were established using the pGIPZ and pInducer10 vectors. The inducible cells were treated with 4 μg/ml Dox for at least 3 days to obtain significant gene knockdown or 0.5 μg/ml Dox for at least 3 days for gene overexpression. The sequences of shRNAs are as following: shMAPK4-G2, GGGTTGGTAACAAAGTGGT, shMAPK4-G4, CGGGAGGAAGACAAGGACG, shPDK1-1: AGGAGATTGTCATAATTGC, shPDK1-2: ATAAGATACTCGTTTCCAG, sheIF4E-1: TTGGAGATCAGCCGCAGGT, sheIF4E-2: GCAACCTCCTGATTAGATT.

### Proliferation assay

Cells were seeded at 2,000 to 8,000 cells per well (cell line dependent) in 500 μl media in a 48-well plate or 1,500 to 5,000 cells per well (cell line dependent) in 200 μl media in a 96-well plate. At the predetermined time points, cell culture was stopped and then fixed with 10% (w/v) formaldehyde for 15 min. Cells were stained with 0.05% (w/v) crystal violet supplemented with 10% (v/v) methanol and 10% (v/v) ethanol for 20 min at room temperature. Approximately 200 to 500 μl 10% acetic acid was added to each well after washing and drying, then absorbance was read at 570 nm, and 0.5 μg/ml Doxycycline was used for induced gene overexpression, and 4 μg/ml Doxycycline was used for induced gene knockdown. When applicable, PI3K inhibitors Alpelisib, AKT inhibitor MK2206, PDK1 inhibitor GSK2334470, and MNK1/2 inhibitors SLV-2436/SEL201 or EFT-508 at the indicated concentrations were added the day after cell seeding and refreshed every 3 days if needed.

### Plate colony formation assay and soft agar assay

For plate colony assay, 2,000 to 8,000 cells/well (cell line dependent) were seeded in 12-well plates in 1 ml media and replaced with fresh media every week. Approximately 10% (w/v) formaldehyde was used to fix cells for 15 min after 2 to 3 weeks culture, and 0.05% (w/v) crystal violet supplemented with 10% methanol and 10% ethanol was used to stain cells for 20 min at room temperature. Then removed the staining solution and washed the plate with distilled water. The plates were then air-dried and scanned using a Canon scanner. Then added 500 μl 10% acetic acid to each well and read the OD absorbance at 570 nm. Soft agar assays were performed as previously described [3,4,18,19]. Full medium containing 0.4% low melting agarose at 37°C was used to mix the cells and then plated onto supporting gel (0.8% agarose); 4 μg/ml or 0.5 μg/ml Dox was used for inducible gene knockdown or inducible overexpression. Soft agar was fixed using 10% acetic acid and 5% methanol, after 3 to 4 weeks of culture. ImageJ software was used to count the Numbers of colonies (with an estimated minimal colony size of 50 to 100 μm). When applicable, PI3K and/or PDK1 inhibitors at the indicated concentrations were added during the initial setup and replenished in fresh media every week.

### Western blotting

RIPA buffer (100 mM NaCl, 0.5% sodium deoxycholate, 0.1% SDS, 50 mM Tris-HCl [pH 8.0], 1% Triton X-100 with 100× protease inhibitors cocktail solution [GenDEPOT, catalog P3100-005] and the phosphatase inhibitors NaF [10 mM] and Na3VO4 [20 mM]) was used to prepare the protein samples. The bicinchoninic acid (BCA) protein assay kit (Thermo Scientific, catalog 23225) was used to quantified the protein sample. The samples were added with 6× loading dye and boiled at 97°C for 7 min. An equal amount of 10 to 20 μg protein per line was used in the following SDS-PAGE and immunoblotting analysis.

### GST-pull down

The GST-MAPK4 fusion protein was expressed, purified, and subjected to pull-down assay as we previously reported [3]. GST-HF protein was used as control. Immunoprecipitation was performed using EZview Red ANTI-FLAG M2 Affinity Gel (Millipore Sigma), as we previously reported [3,4,18–20].

### Xenograft studies

The engineered SUM159 or MDA-MB-231 cells (2 × 10^6^) in Matrigel (1:1) were injected into the fourth mammary fat pad of 8 to 10 weeks old female SCID/beige mice from Envigo. On the day of tumor inoculation, mice began to receive Dox (0.5 mg/ml for inducible overexpression of MAPK4 or control) in 1% sucrose in drinking water. Tumors were monitored every 2 to 3 days, and 0.52 × length × width^2^ was used to calculate the tumor volumes and recorded. Tumors were harvested as indicated and weighed. Sample sizes were determined based on our previous experience with similar studies [3,4,18–20]. No data were excluded from the analyses. No randomization nor blinding data collection was used.

### Statistics

When there were multiple groups in the analysis, one-way or two-way ANOVA followed by Sidak’s multiple comparisons test was performed using GraphPad Prism 9. *P* < 0.05 was considered significant.

## Supporting information

S1 Fig (**A**) qPCR for PDK1 mRNA expression in the engineered SUM159 cells with 0.5 μg/ml Dox-induced overexpression of MAPK4 (iMAPK4) or control (iCtrl), and in engineered HCC1937 and HS578T cells with 4 μg/ml Dox-induced knockdown of MAPK4 (iG2 and iG4) or control (iNT). Quantification data as means ± SD. Western blots on PDK1 protein expression in the (**B**) indicated human TNBC cell lines treated with 100 μg/ml CHX for indicated time (hours) and (**C**) engineered SUM159 and H578T cells with 4 μg/ml Dox-induced knockdown of MAPK4 (iG2, iG4) or control (iNT), also treated with 20 μm MG132 or vehicle control for 4 h. Data are representative of at least 3 independent experiments. The numerical values underlying the figures can be found in S1 Data.
(TIFF)

S2 Fig (**A**) Western blots on engineered HCC1806 and SUM159 cells with 0.5 μg/ml Dox-induced overexpression of MAPK4 (iMAPK4) or control (iCtrl), also with stable knockdown of eIF4e (sh4E-1 and sh4E-2) or control (NT). Western blots on engineered (**B**) SUM159, MDA-MB-468, HCC1395, and HCC1806 cells with 0.5 μg/ml Dox-induced overexpression of MAPK4 (iMAPK4) or control (iCtrl), and (**C**) HS578T, SUM159, and HCC1937 cells with 4 μg/ml Dox-induced knockdown of MAPK4 (iG2 and iG4) or control (iNT). (**D**) co-IP assays showing MAPK4-eIF4E interaction. HEK293T cells were transfected with HA-tagged MAPK4 (MAPK4-HA) and Flag-tagged eIF4E (Flag-eIF4E, left Panel) or Flag/His-tagged MAPK4 (MAPK4-FH) and HA-tagged eIF4E (HA-eIF4E, Right Panel), and 48 h later, cell lysates were prepared for the immunoprecipitation using anti-FLAG M2 affinity gel followed by western blots using indicated antibodies. (**E**) GST pull-down assay showing purified GST-MAPK4 binding with endogenous eIF4E in MDA-MB-231, HS578T, and HCC1937 cell lysates. Coomassie blue staining (right panel) revealed a major band of around 100 kDa and 30 kDa in the purified GST-MAPK4 and GST-FH proteins, respectively. MW, molecular weight. FH: 2× FLAG and 10× His tag. Data are representative of at least 3 independent experiments.
(TIFF)

S3 Fig Western blots on (**A**) engineered HCC1806 and SUM159 cells with 0.5 μg/ml Dox-induced overexpression of MAPK4 (iMAPK4) or control (iCtrl) treated with MNK1/2 inhibitors SLV-2436 (2 μm), eFT-508 (1 μm), or DMSO control for 24 h and (B) WT and *MAPK4*-KO SUM159 and MDA-MB-231 cells treated with MNK1/2 inhibitors SLV-2436 (2 μm), eFT-508 (1 μm), or DMSO control for 24 h. Proliferation assays on (**C**) engineered HCC1806 and SUM159 cells with 0.5 μg/ml Dox-induced overexpression of MAPK4 (iMAPK4) or control (iCtrl) and (**D**) WT and *MAPK4*-KO MDA-MB-231 cells treated with MNK1/2 inhibitors SLV-2436 (2 μm), eFT-508 (1 μm), or DMSO control. Quantification data as means ± SD. Data are representative of at least 3 independent experiments. The numerical values underlying the figures can be found in S1 Data.
(TIFF)

S4 Fig Plate colony formation assays on the WT vs. *MAPK4*-knockout (KO, #2) vs. *MAPK4*-knockout with rescued MAPK4 expression (KO+iMAPK4) SUM159 cells. The cells were also treated with 0.5 μg/ml Dox, and DMSO control vs. PDK1 inhibitor GSK2334470 (2 μm) vs. PI3K inhibitor Alpelisib (1 μm) vs. both inhibitors. Quantification data as means ± SD. *P* values by two-way ANOVA followed by Sidak’s multiple comparisons. ***P ≤ 0.001, ****P ≤ 0.0001. Data are representative of at least 3 independent experiments. The numerical values underlying the figures can be found in S1 Data.
(TIFF)

S5 Fig Western blots on PDK1 protein expression in (**A**) the engineered H157 cells with 4 μg/ml Dox-induced knockdown of MAPK4 (iG2 and iG4) or control (iNT), (**B**) parental vs. *MAPK4*-KO H1299 and HCT116 cells, and (**C**) DU145 cells with overexpression of MAPK4 vs. control (Ctrl). Data are representative of at least 2–3 independent experiments.
(TIFF)

S1 Raw Images Uncropped western blots, gel blots for Figs 1A, 1B, 1C, 1D, 2A, 3A, 5A, 5E, 8A, 8B, 8D, S1B, S1C, S2A, S2B, S2C, S2D, S2E, S3A, S3B and S5. (PDF)

S1 Data Numerical data for Figs 2B, 2C, 2D, 2E, 2F, 3B, 3C, 3D, 3E, 3F, 4A, 4B, 5B, 5C, 5D, 5F, 5G, 6A, 6B, 7A, 7B, 7C, 8C, 8E, S1A, S3C, S3D and S4. (XLSX)

## Abbreviations

BCA: bicinchoninic acid
CHX: cycloheximide
MAPK4: mitogen-activated protein kinase 4
PDK1: phosphoinositide-dependent kinase-1
SGK: serum and glucocorticoid-inducible kinase
TNBC: triple-negative breast cancer
WT: wild type
