# Supplementary figures and images for: Cooperative activation of PDK1 and AKT by MAPK4 enhances cancer growth and resistance to therapy
Source: PLoS Biol. 2023 Aug 2;21(8):e3002227. doi: 10.1371/journal.pbio.3002227 (PMC10395914; doi:10.1371/journal.pbio.3002227)

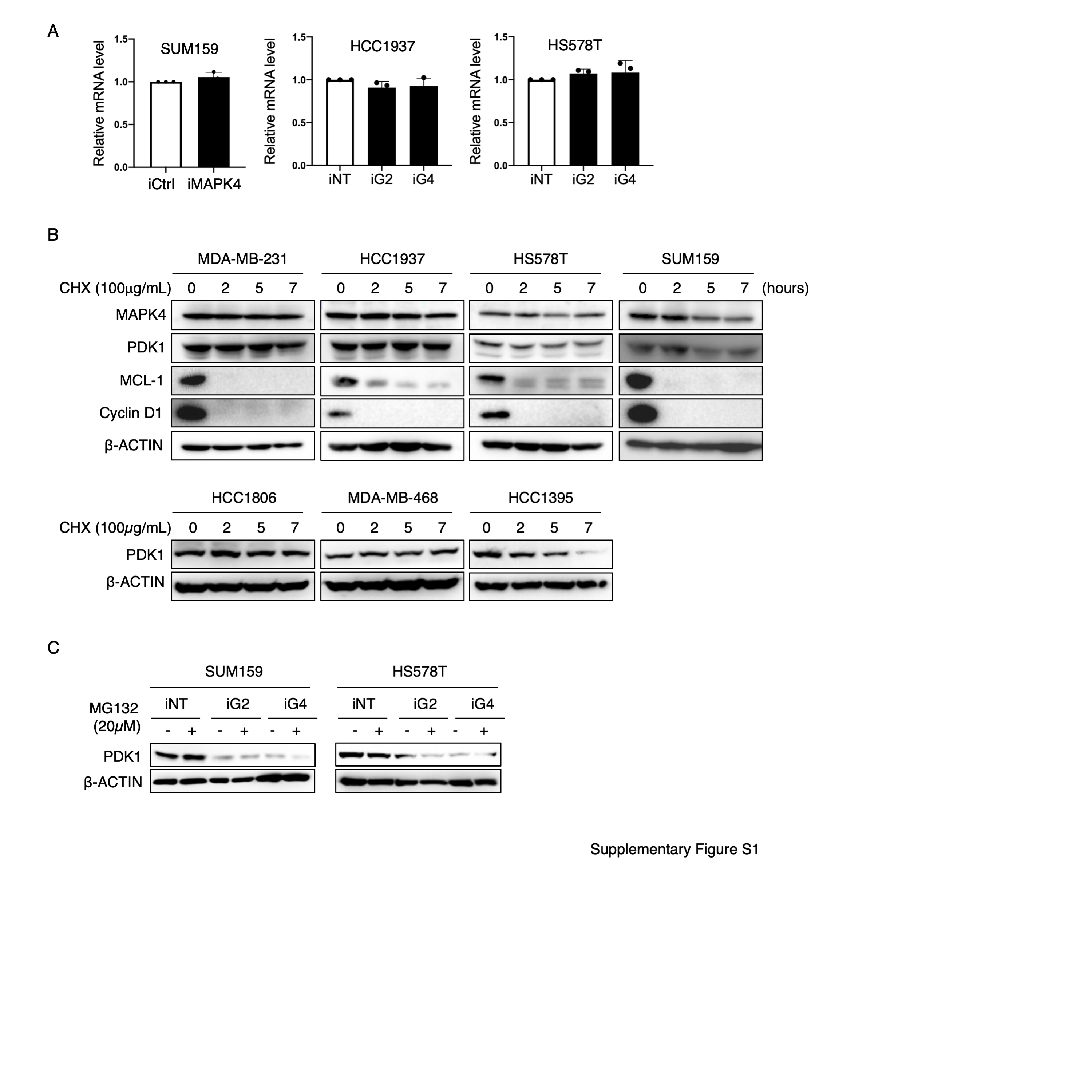

Supplement: S1 Fig — (A) qPCR for PDK1 mRNA expression in the engineered SUM159 cells with 0.5 μg/ml Dox-induced overexpression of MAPK4 (iMAPK4) or control (iCtrl), and in engineered HCC1937 and HS578T cells with 4 μg/ml Dox-induced knockdown of MAPK4 (iG2 and iG4) or control (iNT). Quantification data as means ± SD. Western blots on PDK1 protein expression in the (B) indicated human TNBC cell lines treated with 100 μg/ml CHX for indicated time (hours) and (C) engineered SUM159 and H578T cells with 4 μg/ml Dox-induced knockdown of MAPK4 (iG2, iG4) or control (iNT), also treated with 20 μm MG132 or vehicle control for 4 h. Data are representative of at least 3 independent experiments. The numerical values underlying the figures can be found in S1 Data. (TIFF) [file pbio.3002227.s001.tiff]

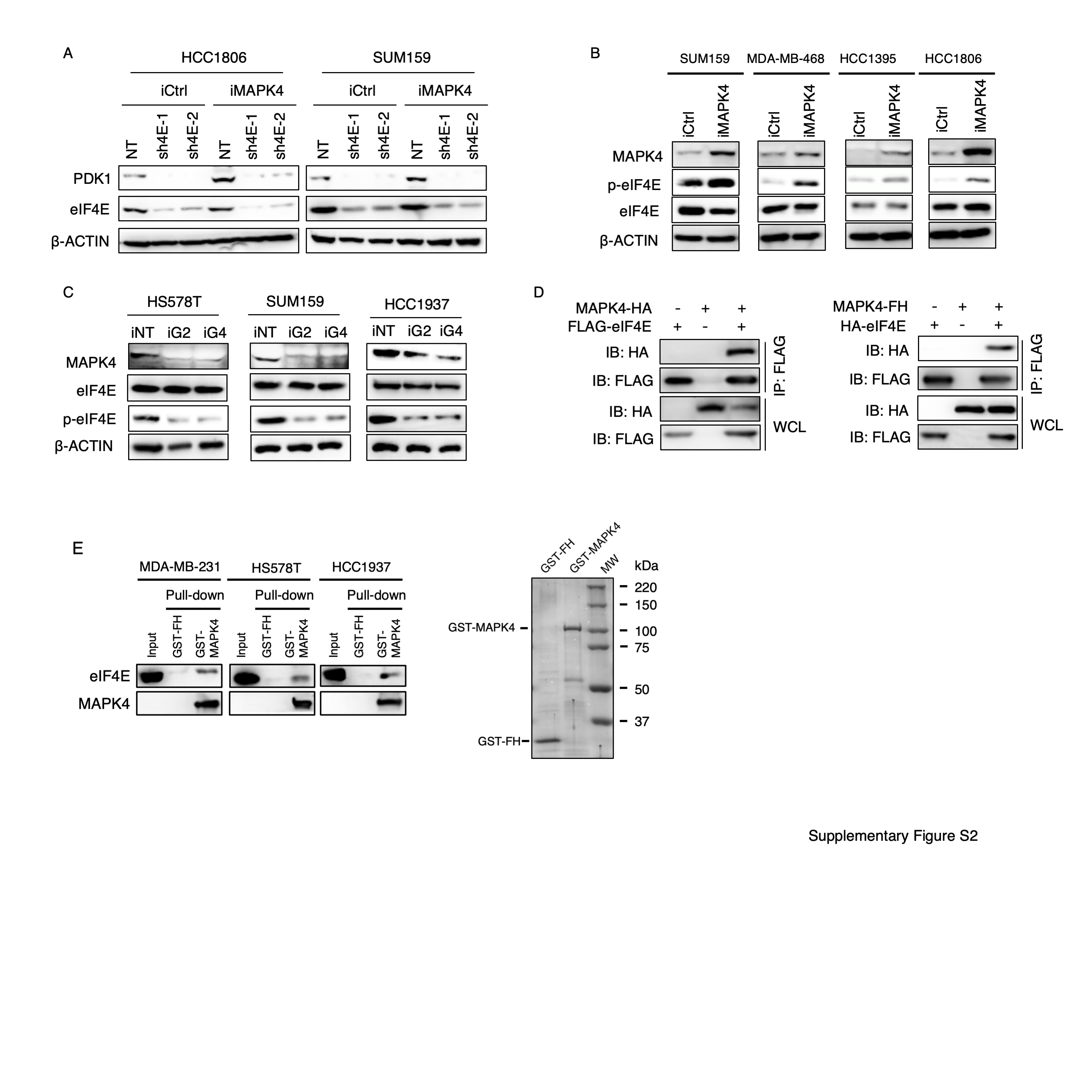

Supplement: S2 Fig — (A) Western blots on engineered HCC1806 and SUM159 cells with 0.5 μg/ml Dox-induced overexpression of MAPK4 (iMAPK4) or control (iCtrl), also with stable knockdown of eIF4e (sh4E-1 and sh4E-2) or control (NT). Western blots on engineered (B) SUM159, MDA-MB-468, HCC1395, and HCC1806 cells with 0.5 μg/ml Dox-induced overexpression of MAPK4 (iMAPK4) or control (iCtrl), and (C) HS578T, SUM159, and HCC1937 cells with 4 μg/ml Dox-induced knockdown of MAPK4 (iG2 and iG4) or control (iNT). (D) co-IP assays showing MAPK4-eIF4E interaction. HEK293T cells were transfected with HA-tagged MAPK4 (MAPK4-HA) and Flag-tagged eIF4E (Flag-eIF4E, left Panel) or Flag/His-tagged MAPK4 (MAPK4-FH) and HA-tagged eIF4E (HA-eIF4E, Right Panel), and 48 h later, cell lysates were prepared for the immunoprecipitation using anti-FLAG M2 affinity gel followed by western blots using indicated antibodies. (E) GST pull-down assay showing purified GST-MAPK4 binding with endogenous eIF4E in MDA-MB-231, HS578T, and HCC1937 cell lysates. Coomassie blue staining (right panel) revealed a major band of around 100 kDa and 30 kDa in the purified GST-MAPK4 and GST-FH proteins, respectively. MW, molecular weight. FH: 2× FLAG and 10× His tag. Data are representative of at least 3 independent experiments. (TIFF) [file pbio.3002227.s002.tiff]

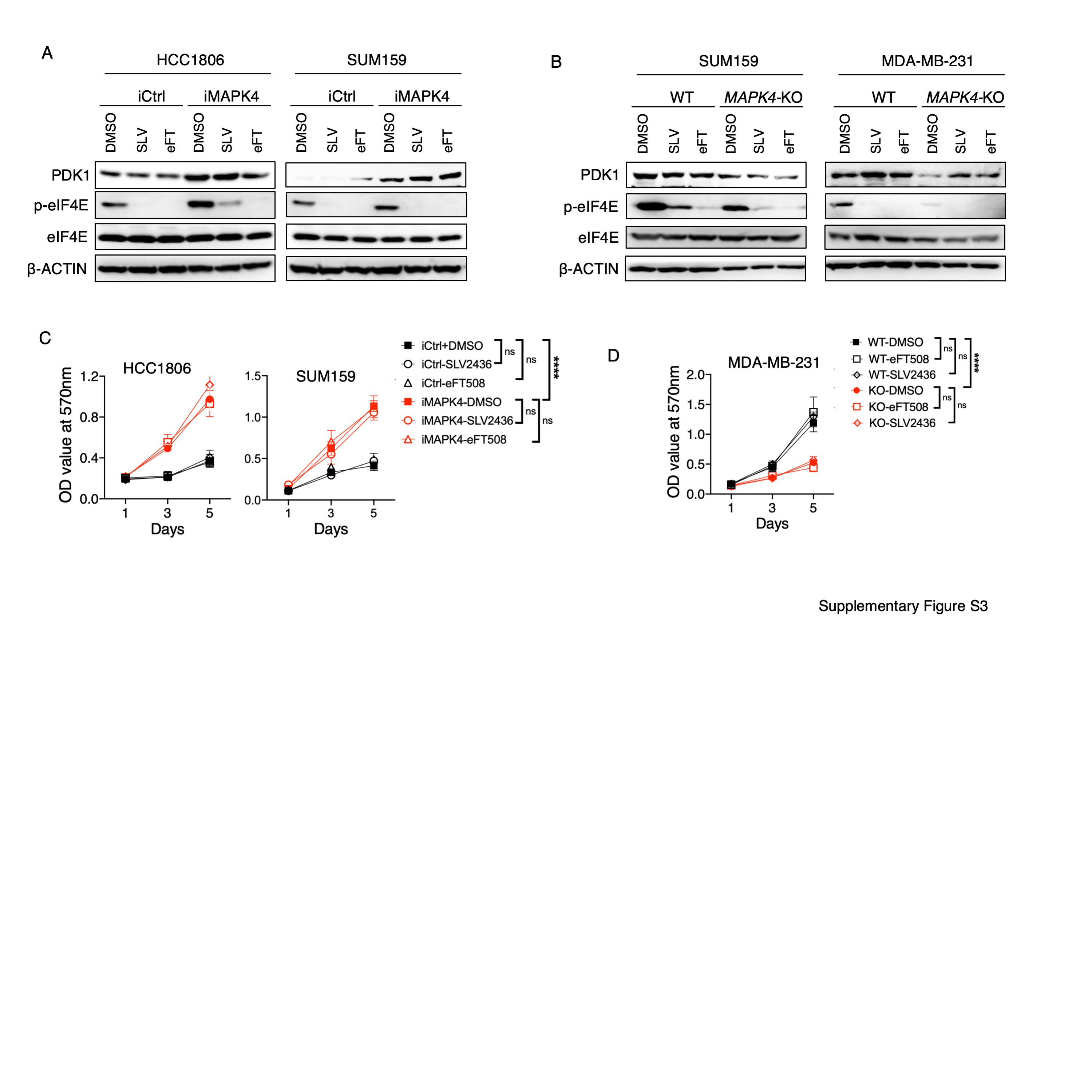

Supplement: S3 Fig — Western blots on (A) engineered HCC1806 and SUM159 cells with 0.5 μg/ml Dox-induced overexpression of MAPK4 (iMAPK4) or control (iCtrl) treated with MNK1/2 inhibitors SLV-2436 (2 μm), eFT-508 (1 μm), or DMSO control for 24 h and (B) WT and MAPK4-KO SUM159 and MDA-MB-231 cells treated with MNK1/2 inhibitors SLV-2436 (2 μm), eFT-508 (1 μm), or DMSO control for 24 h. Proliferation assays on (C) engineered HCC1806 and SUM159 cells with 0.5 μg/ml Dox-induced overexpression of MAPK4 (iMAPK4) or control (iCtrl) and (D) WT and MAPK4-KO MDA-MB-231 cells treated with MNK1/2 inhibitors SLV-2436 (2 μm), eFT-508 (1 μm), or DMSO control. Quantification data as means ± SD. Data are representative of at least 3 independent experiments. The numerical values underlying the figures can be found in S1 Data. (TIFF) [file pbio.3002227.s003.tiff]

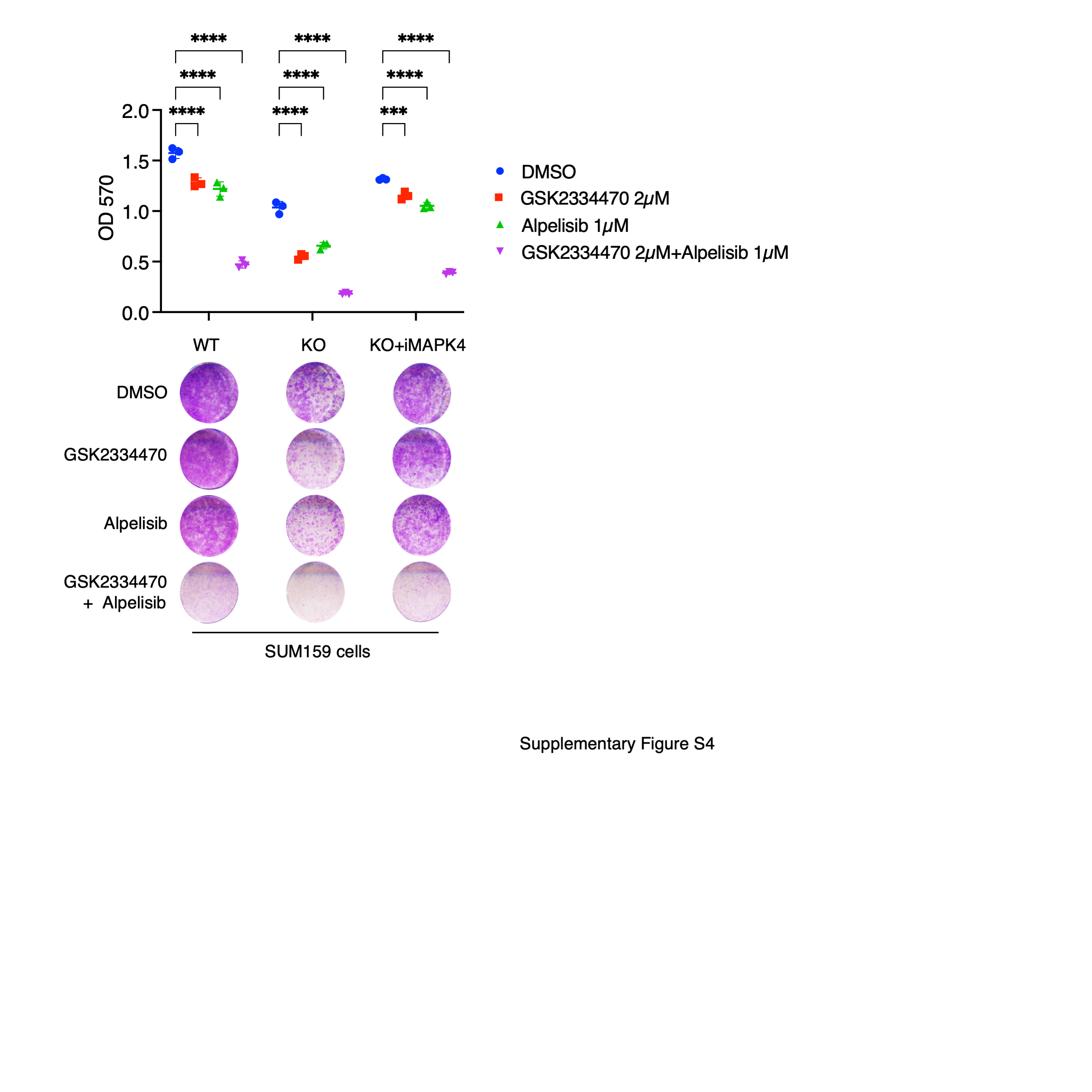

Supplement: S4 Fig — Plate colony formation assays on the WT vs. MAPK4-knockout (KO, #2) vs. MAPK4-knockout with rescued MAPK4 expression (KO+iMAPK4) SUM159 cells. The cells were also treated with 0.5 μg/ml Dox, and DMSO control vs. PDK1 inhibitor GSK2334470 (2 μm) vs. PI3K inhibitor Alpelisib (1 μm) vs. both inhibitors. Quantification data as means ± SD. P values by two-way ANOVA followed by Sidak’s multiple comparisons. ***P ≤ 0.001, ****P ≤ 0.0001. Data are representative of at least 3 independent experiments. The numerical values underlying the figures can be found in S1 Data. (TIFF) [file pbio.3002227.s004.tiff]

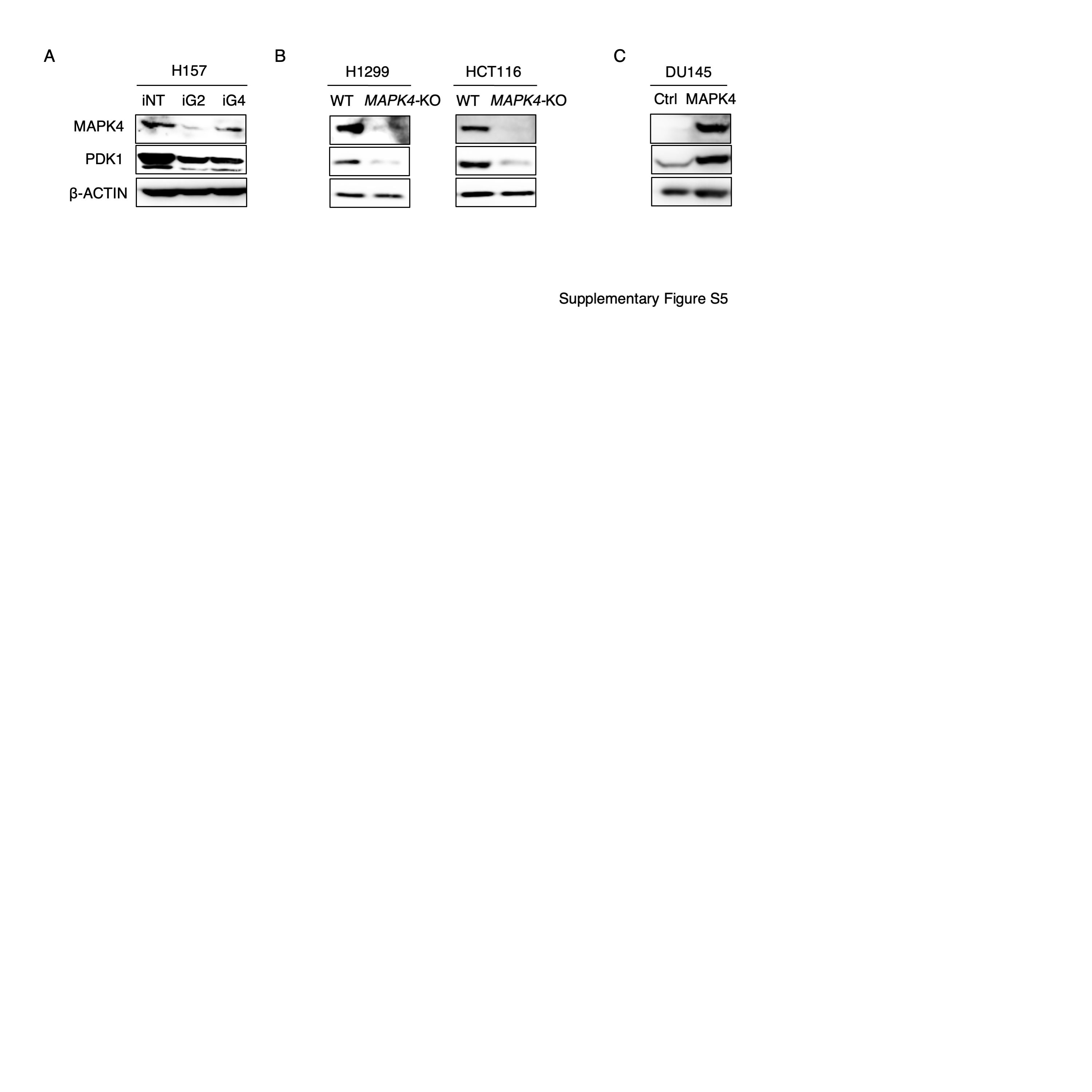

Supplement: S5 Fig — Western blots on PDK1 protein expression in (A) the engineered H157 cells with 4 μg/ml Dox-induced knockdown of MAPK4 (iG2 and iG4) or control (iNT), (B) parental vs. MAPK4-KO H1299 and HCT116 cells, and (C) DU145 cells with overexpression of MAPK4 vs. control (Ctrl). Data are representative of at least 2–3 independent experiments. (TIFF) [file pbio.3002227.s005.tiff]
