# Supplementary material for: Cooperative activation of PDK1 and AKT by MAPK4 enhances cancer growth and resistance to therapy
Source: PLoS Biol. 2023 Aug 2;21(8):e3002227. doi: 10.1371/journal.pbio.3002227 (PMC10395914; doi:10.1371/journal.pbio.3002227)
Supplement: S1 Raw Images — (PDF) [file pbio.3002227.s006.pdf]

Figure 1 A

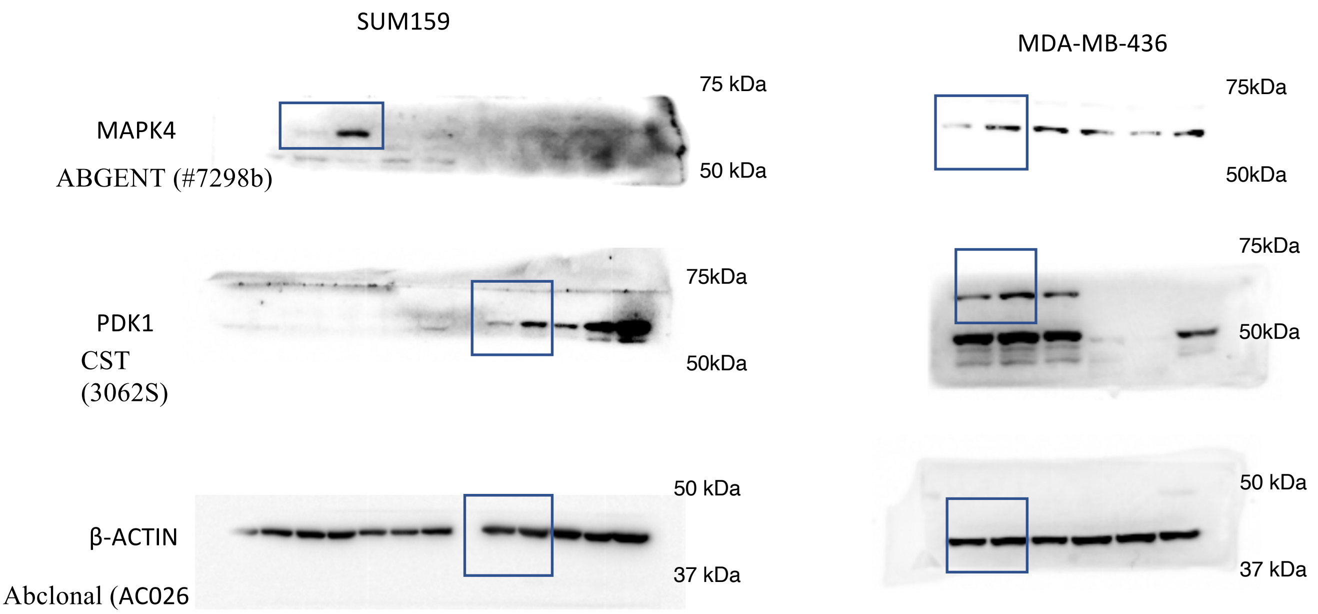

Figure 1 A

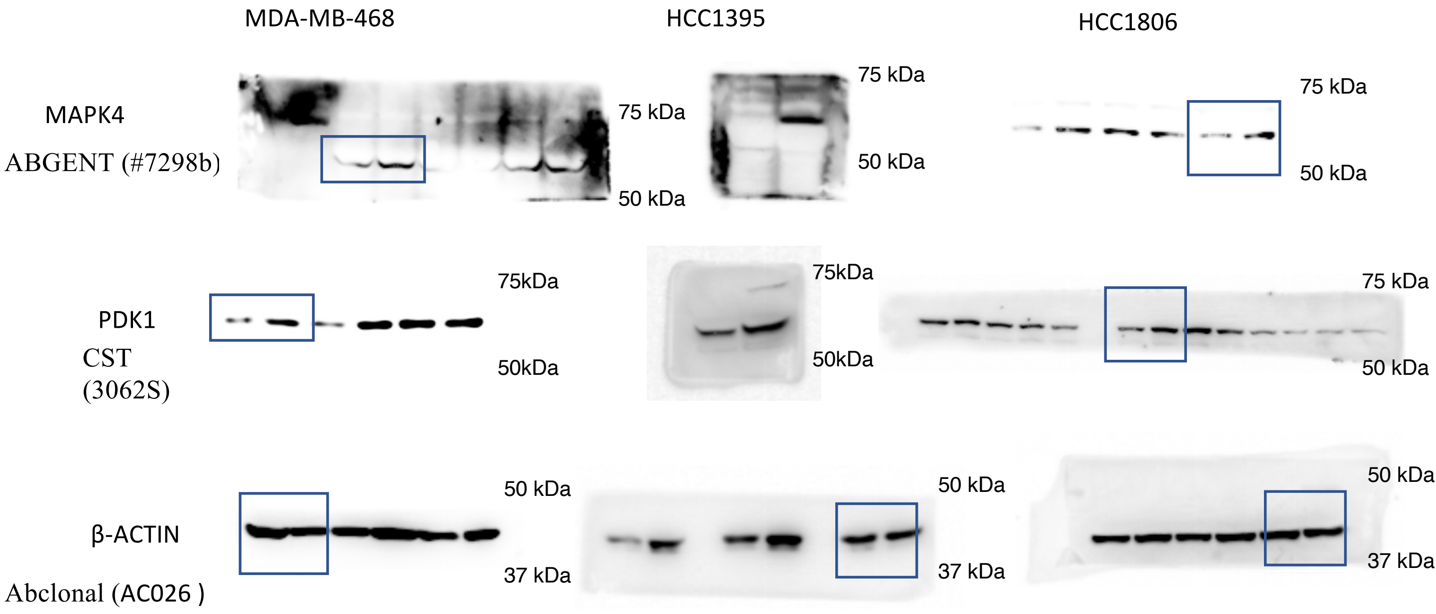

Figure 1 B

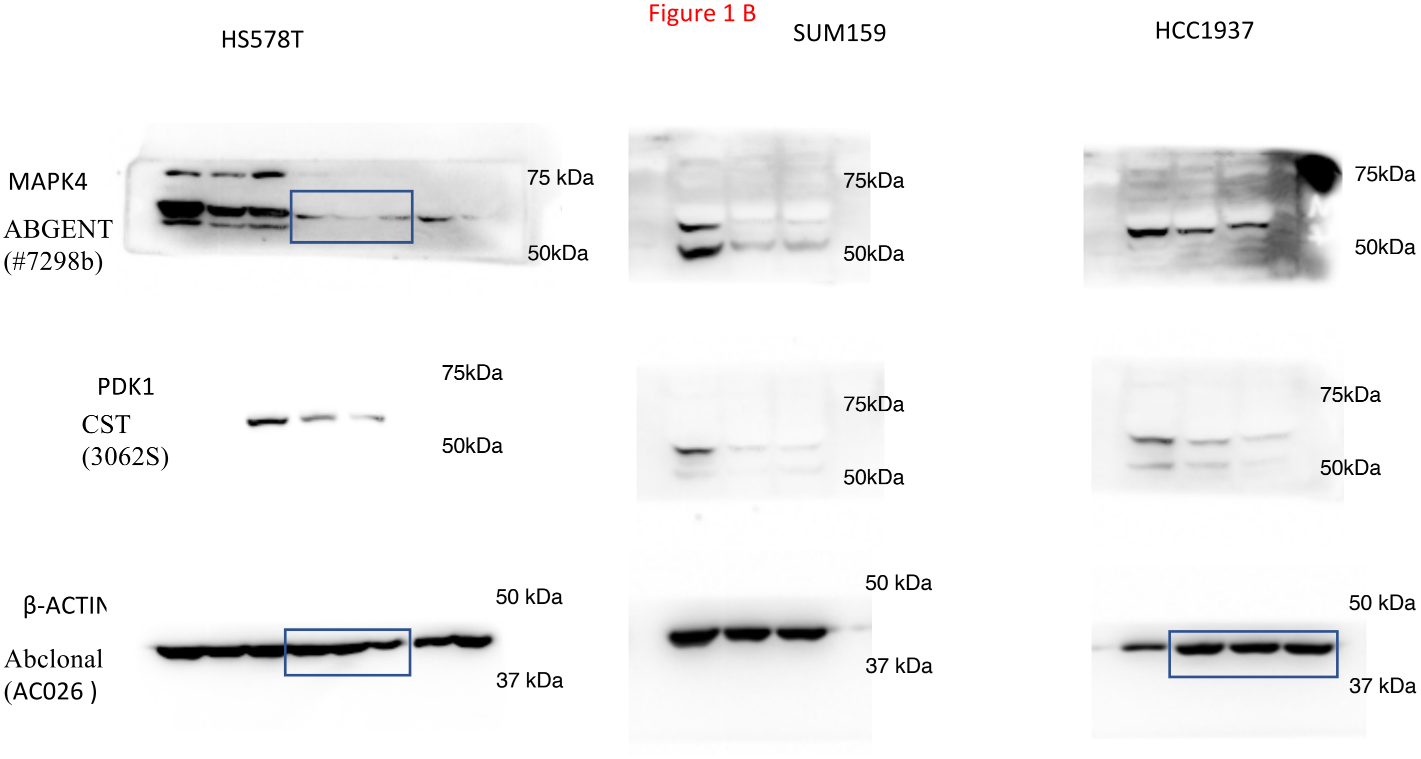

Figure 1 C

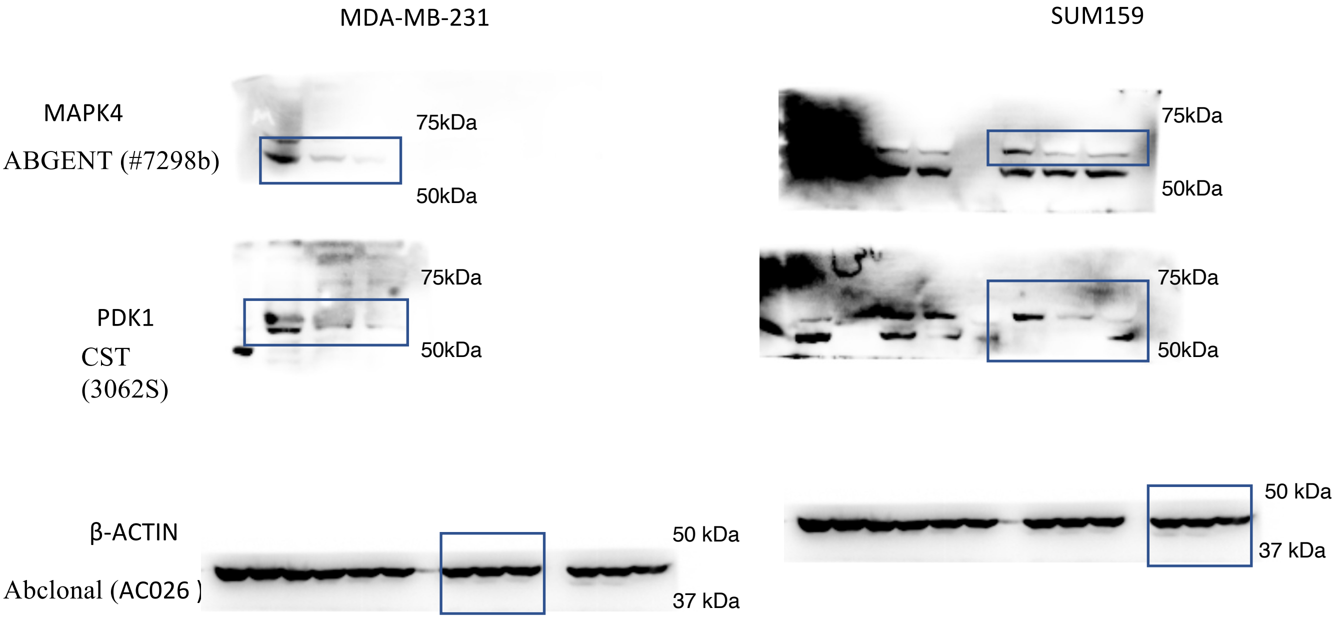

Figure 1 D

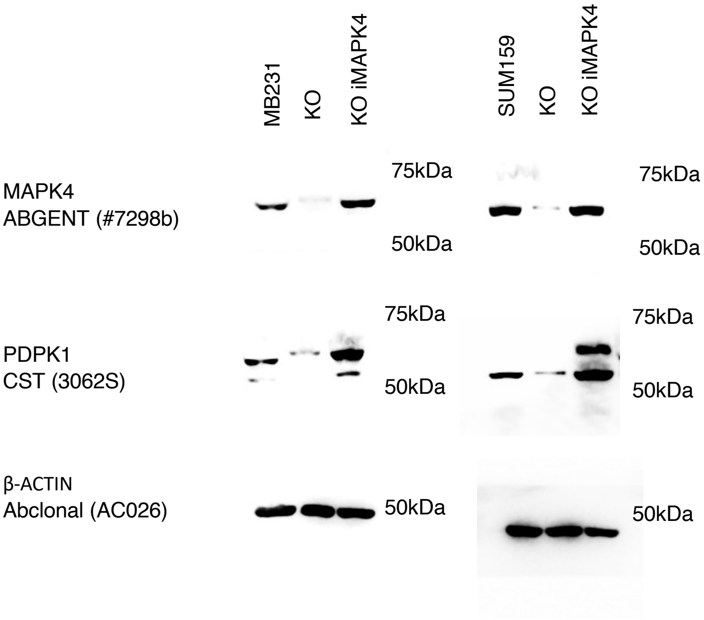
MB231
KO
KO iMAPK4
SUM159
KO
KO iMAPK4
MAPK4
ABGENT (#7298b)
75kDa
50kDa
75kDa
50kDa
PDPK1
CST (3062S)
75kDa
50kDa
75kDa
50kDa
β-ACTIN
Abclonal (AC026)
50kDa
50kDa

Figure 2A SUM159

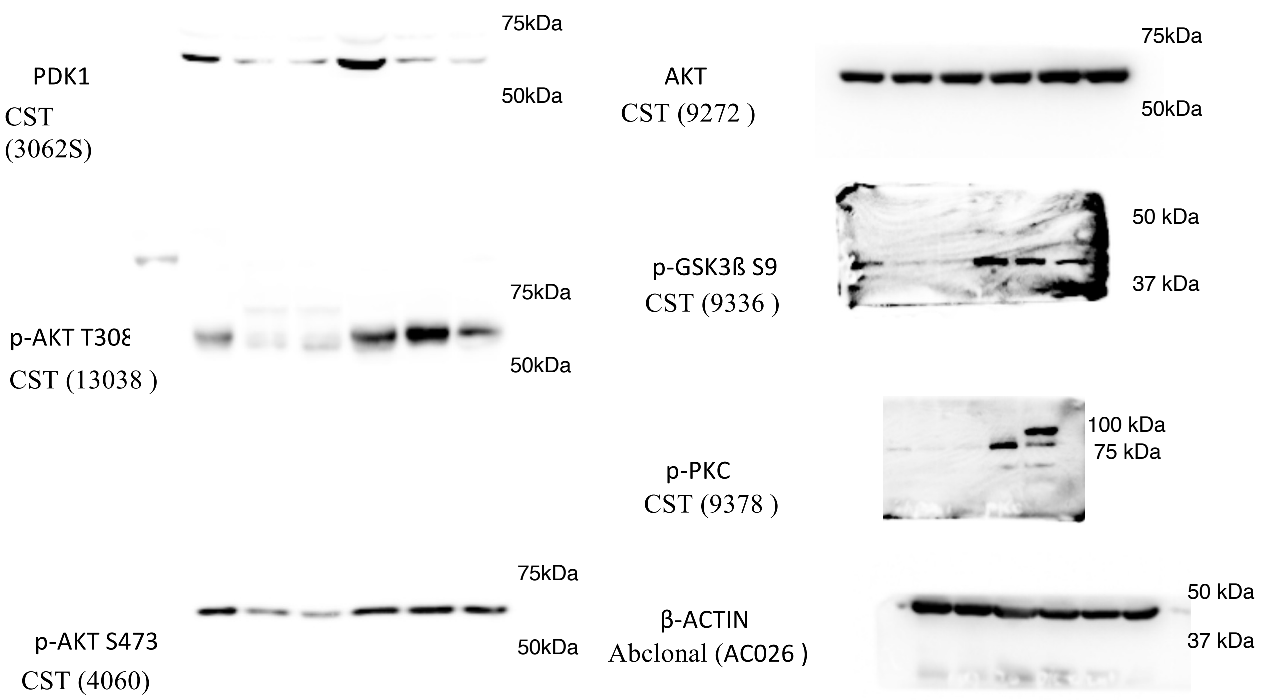

Figure 2A HCC1806

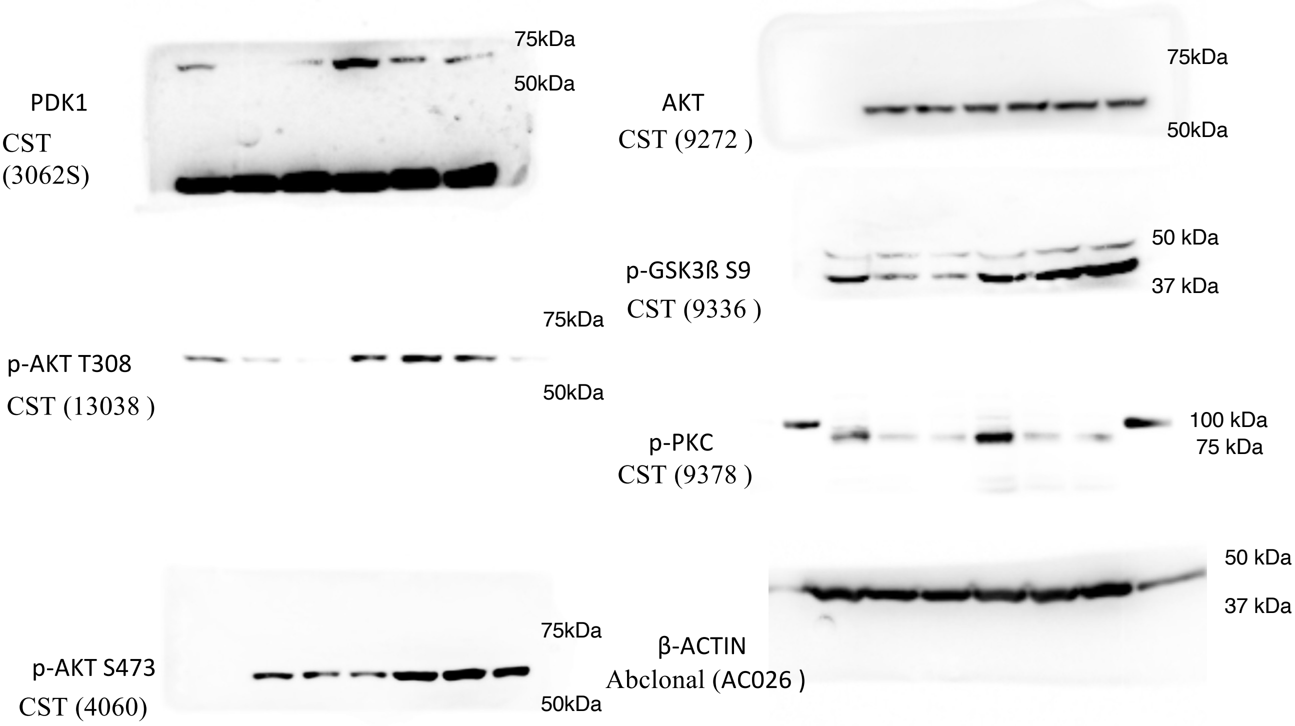

Figure 3A

SUM159

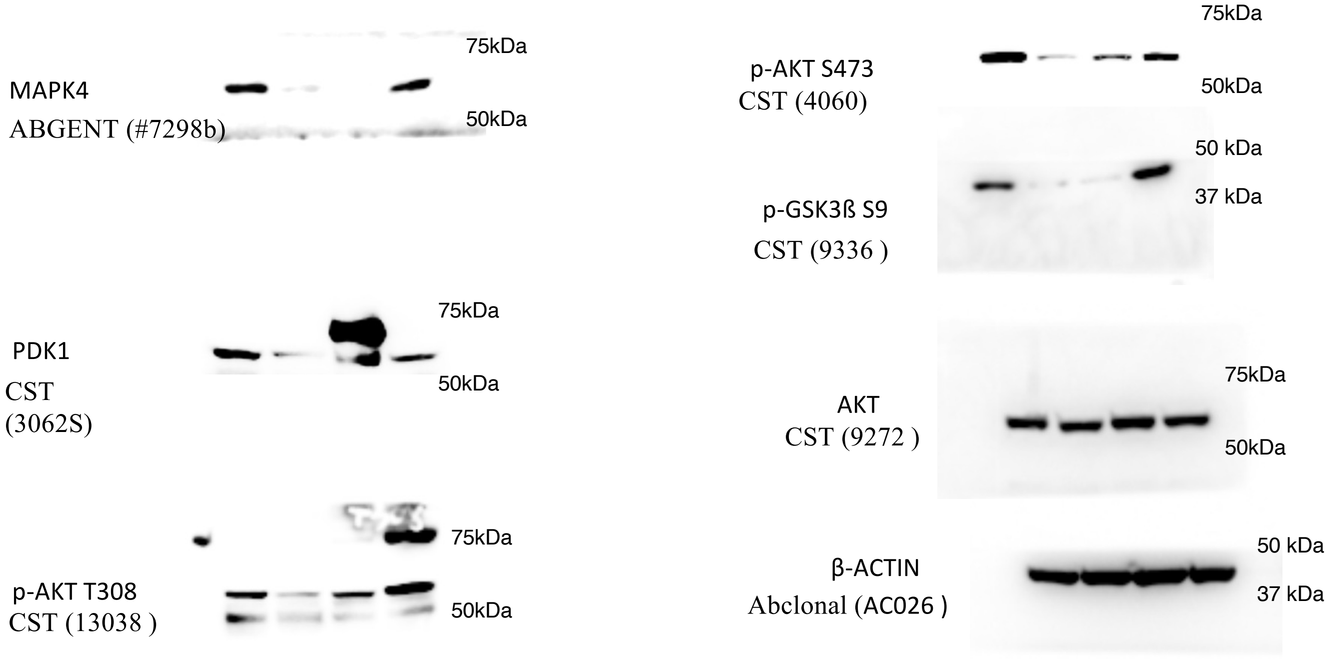

Figure 3A

MDA-MB-231

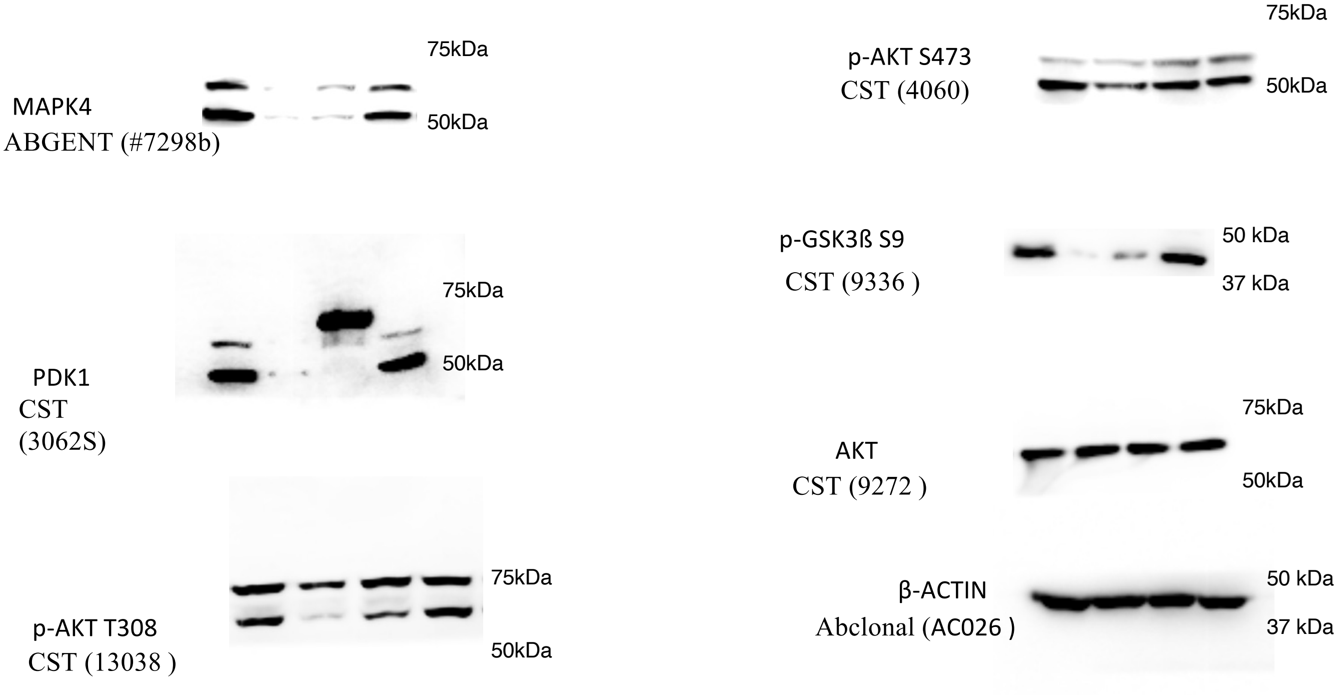

Figure 5A SUM159

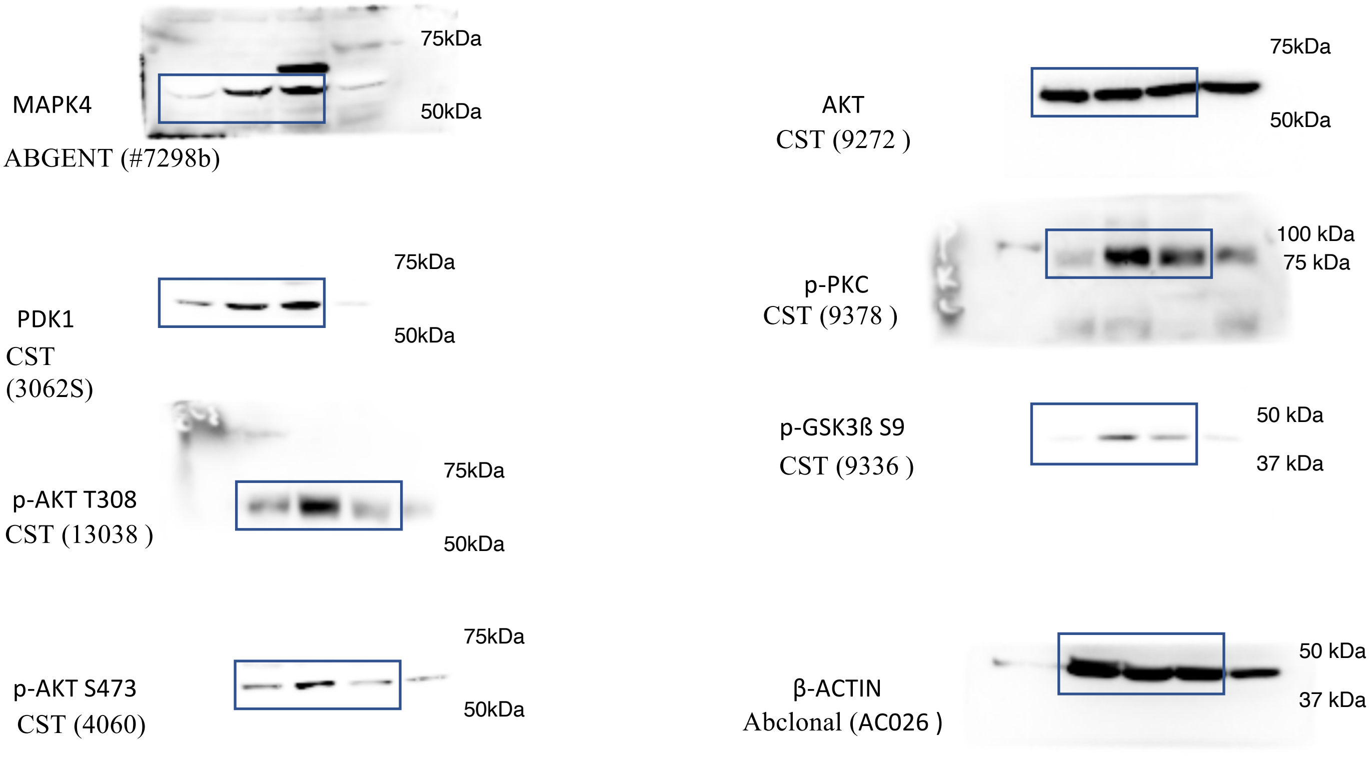

Figure 5A HCC1806

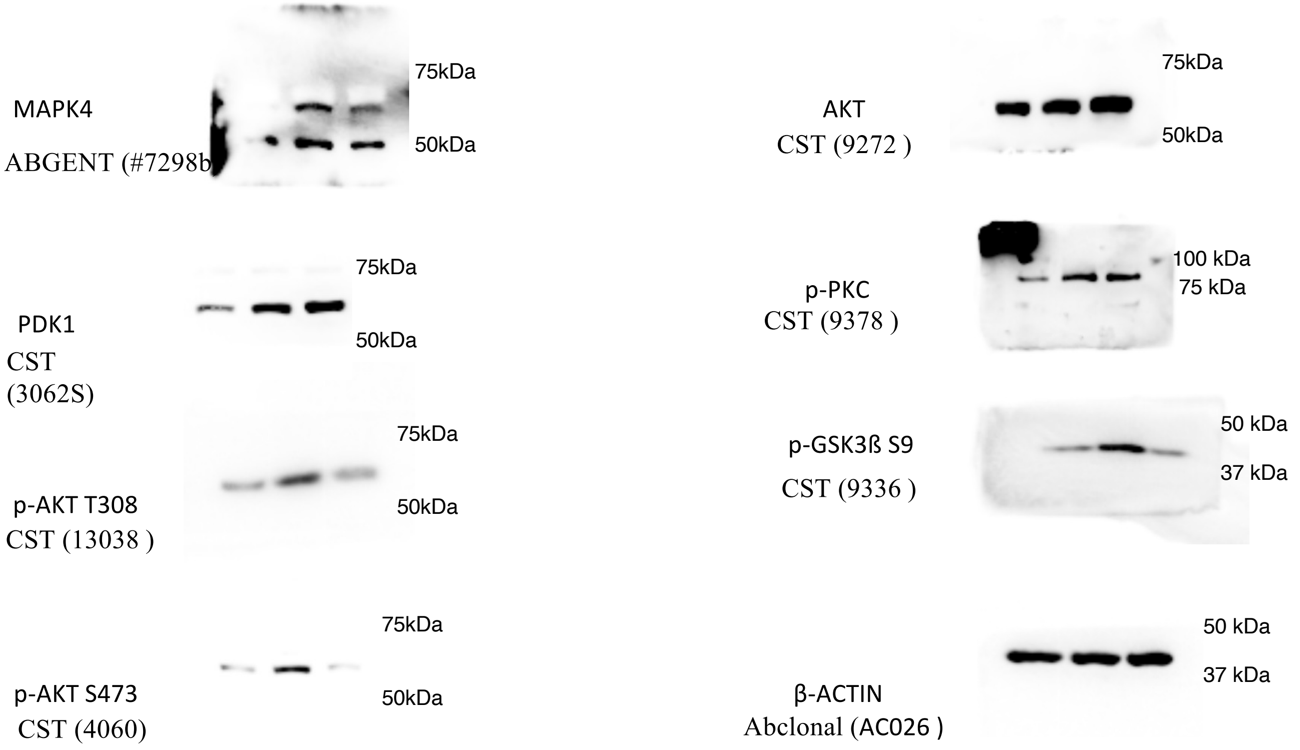

Figure 5E

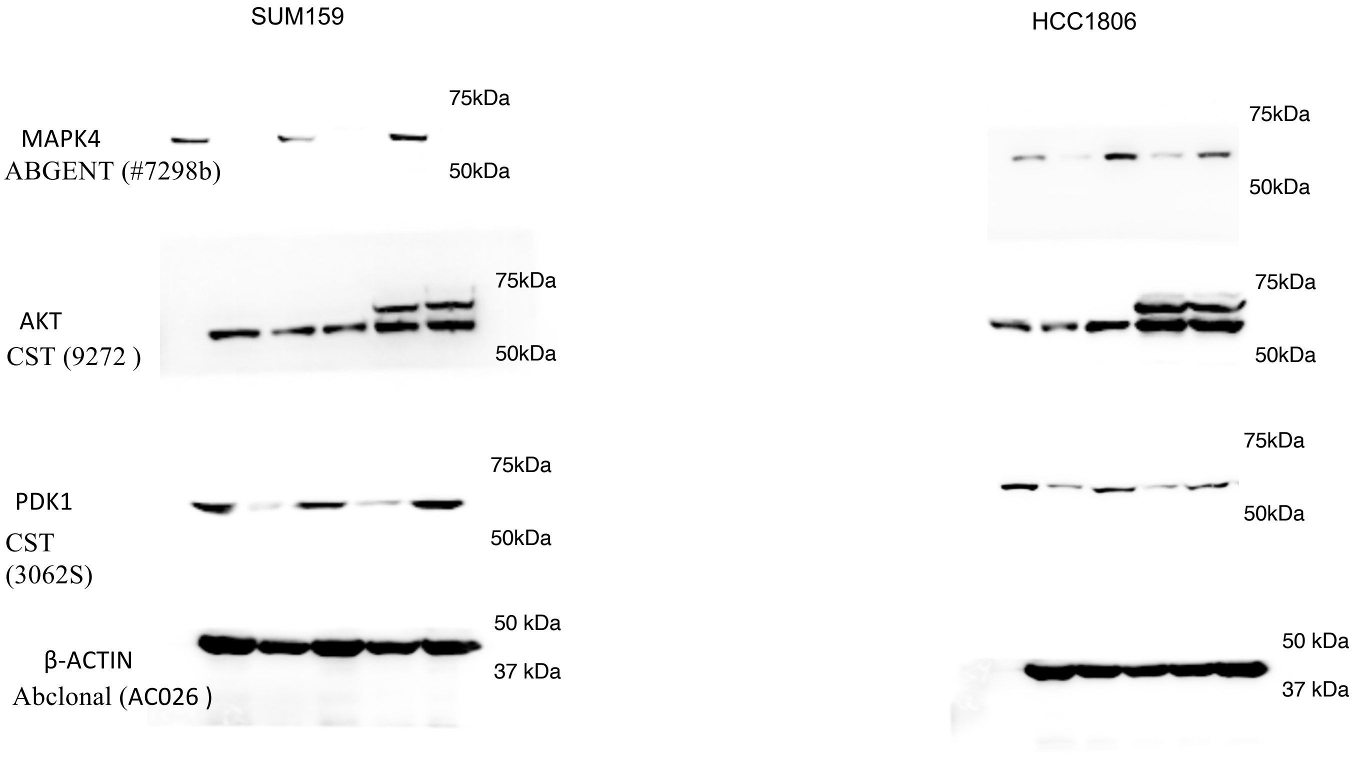

SUM159
HCC1806
MAPK4
ABGENT (#7298b)
75kDa
50kDa
AKT
CST (9272)
75kDa
50kDa
PDK1
CST
(3062S)
75kDa
50kDa
β-ACTIN
Abclonal (AC026)
50 kDa
37 kDa

Figure 8A SUM159

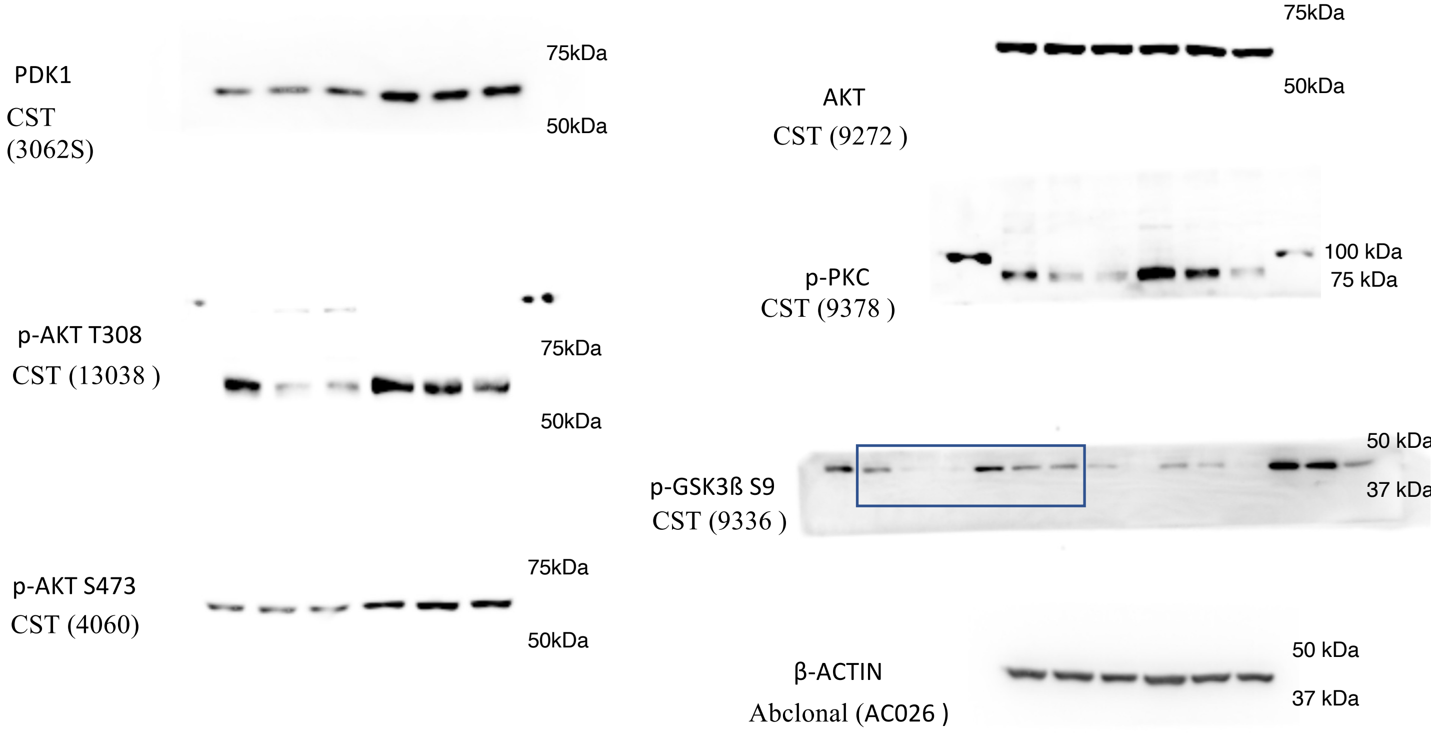

Figure 8A HCC1806

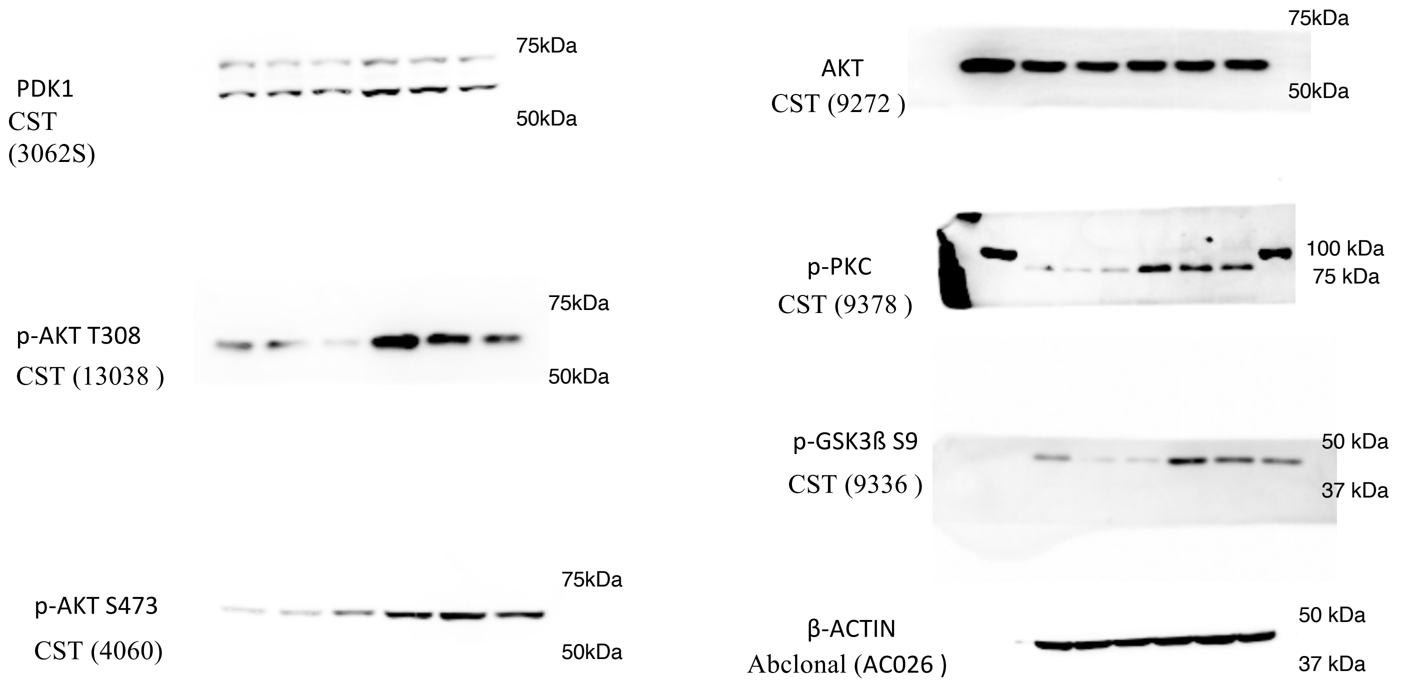

Figure 8B SUM159

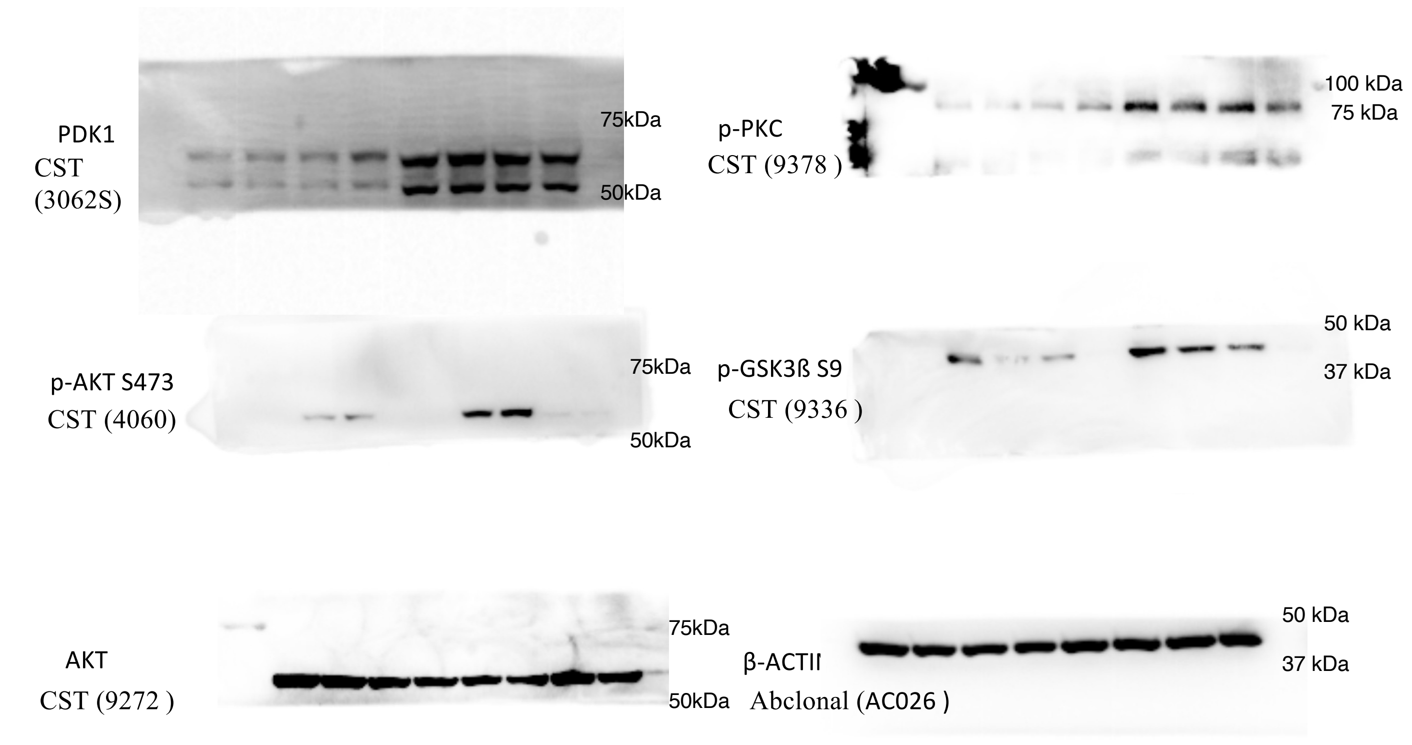

Figure 8B HCC1806

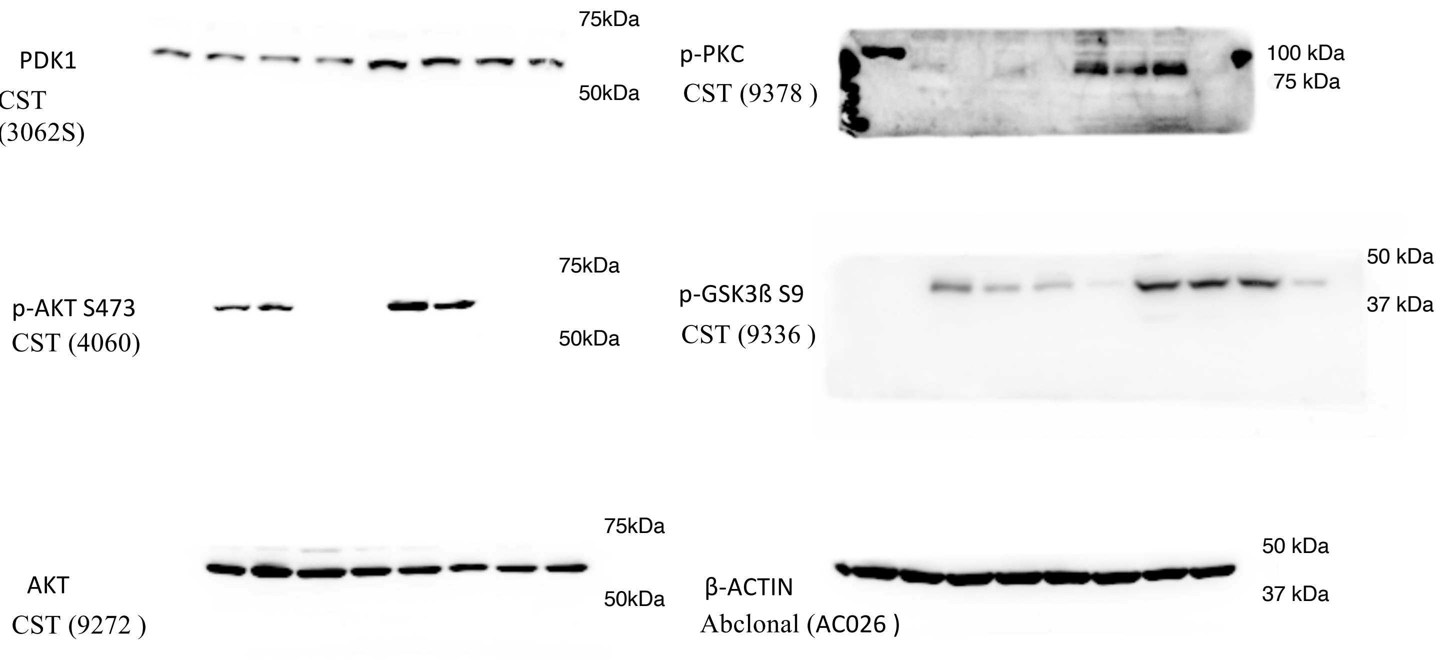

Figure 8D

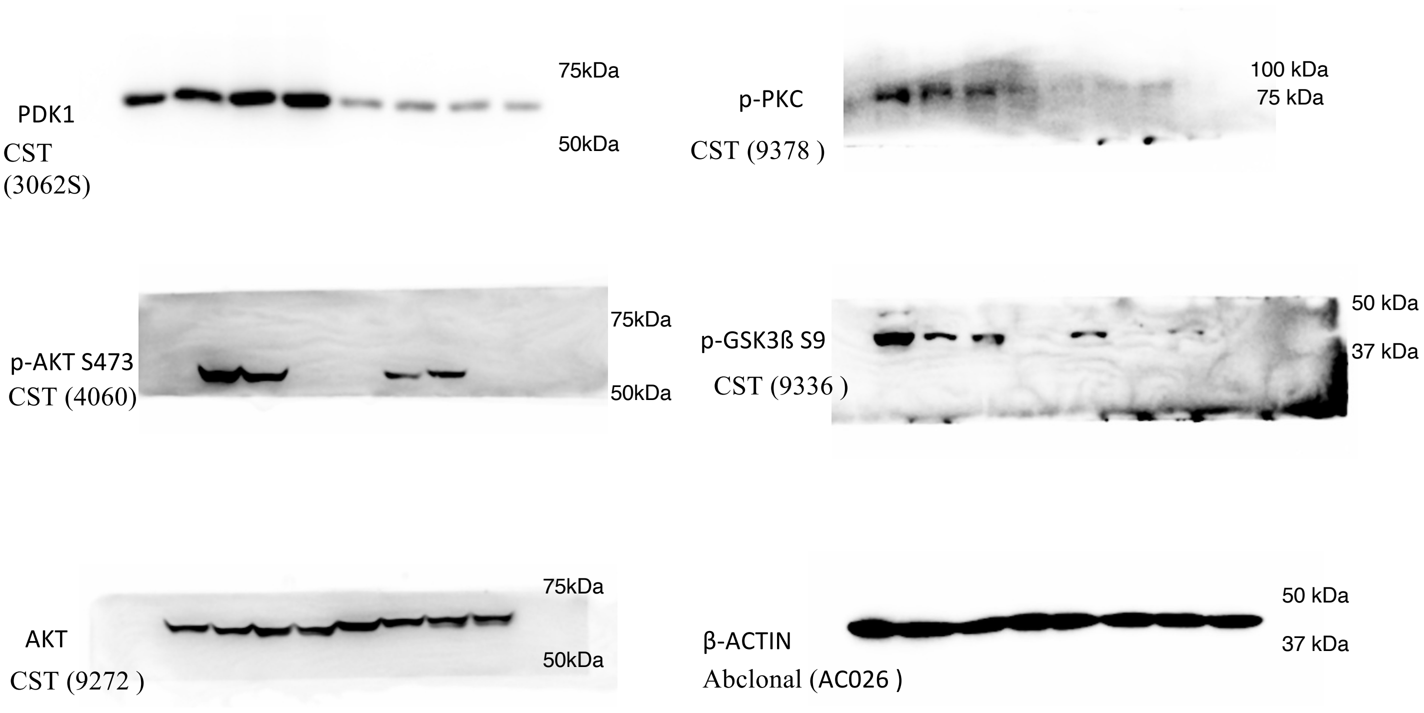
PDK1
CST (3062S)
75kDa
50kDa
p-PKC
CST (9378)
100 kDa
75 kDa
p-AKT S473
CST (4060)
75kDa
50kDa
p-GSK3ß S9
CST (9336)
50 kDa
37 kDa
AKT
CST (9272)
75kDa
50kDa
β-ACTIN
Abclonal (AC026)
50 kDa
37 kDa

Figure S1 B

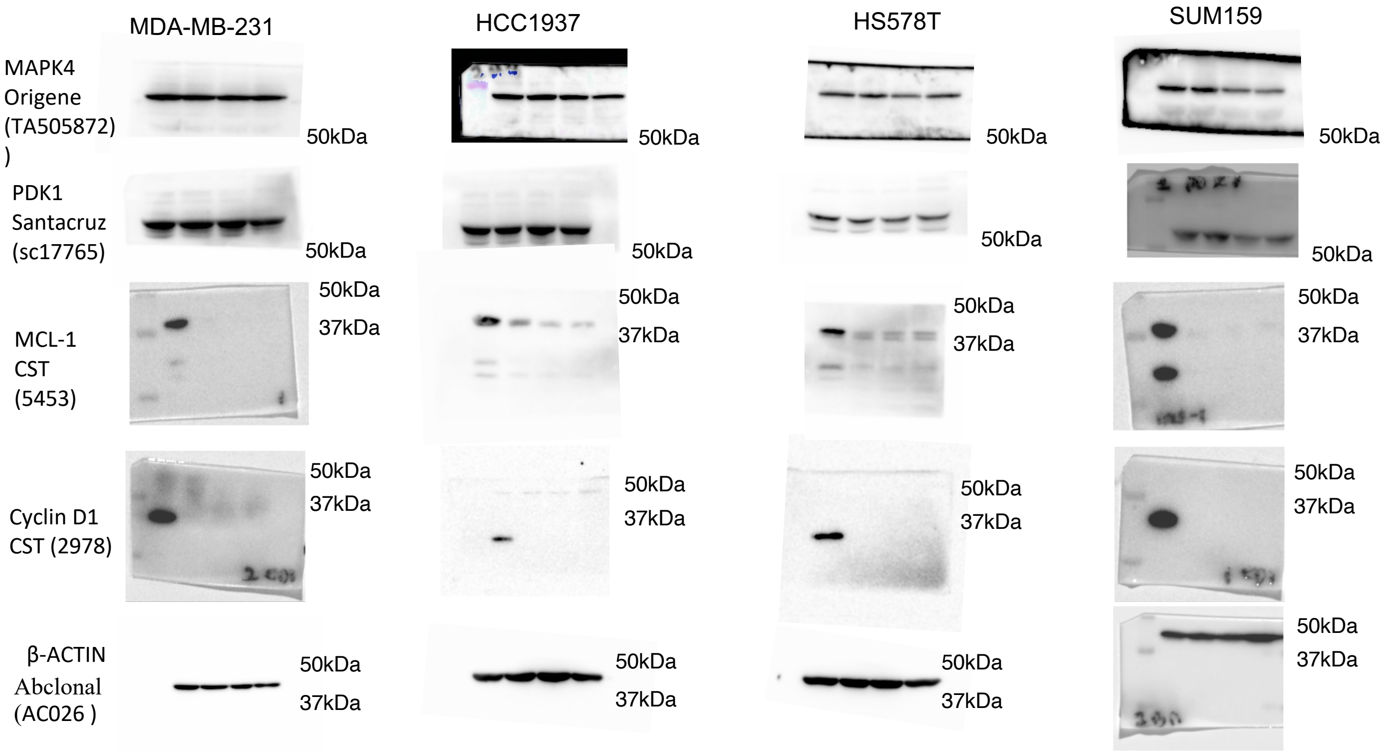

Figure S1 B

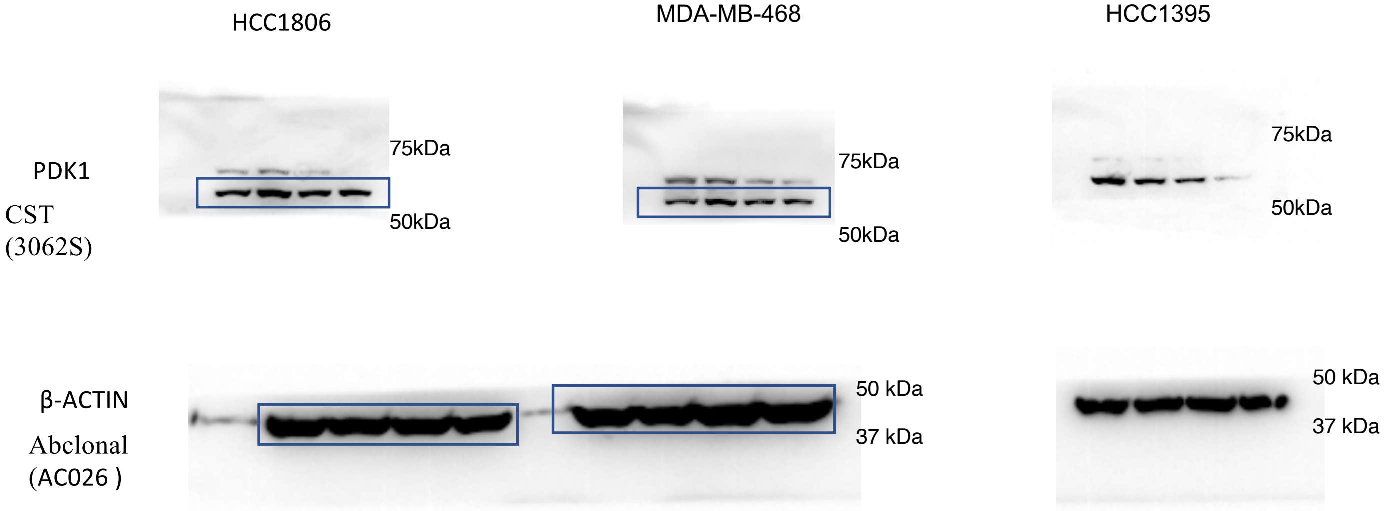

Figure S1C

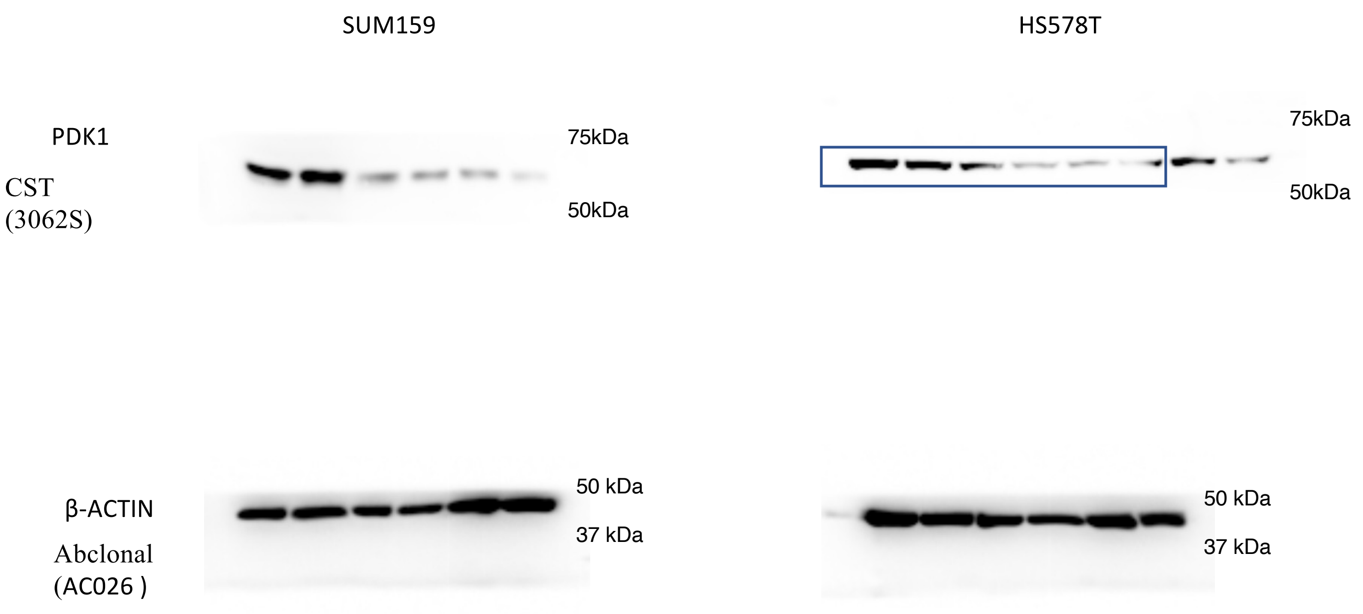

Figure S2A

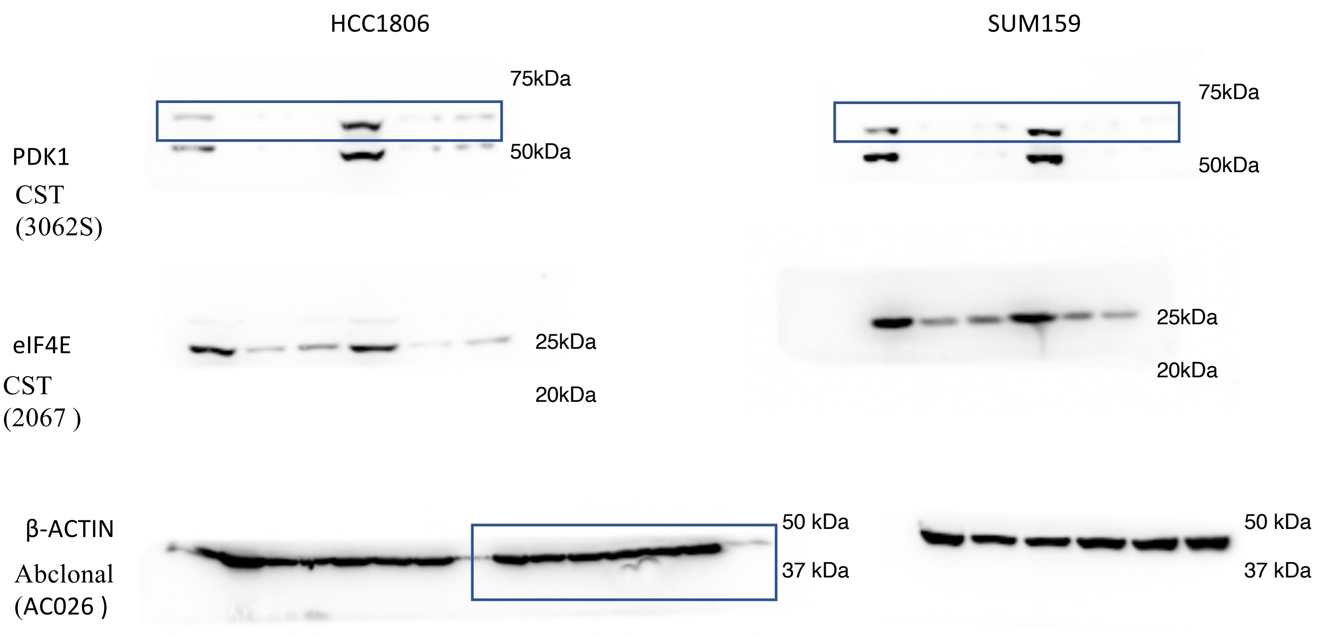

Figure S2 B

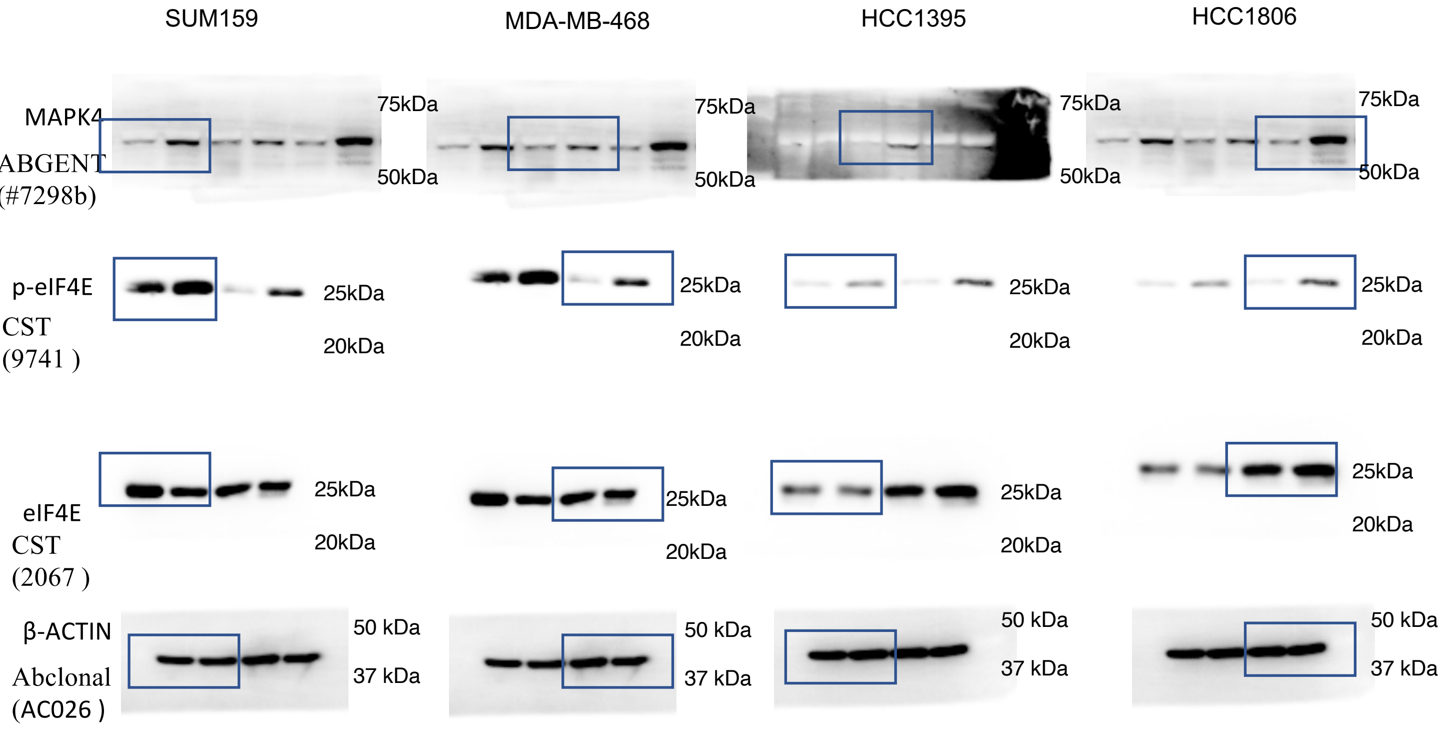

Figure S2 C

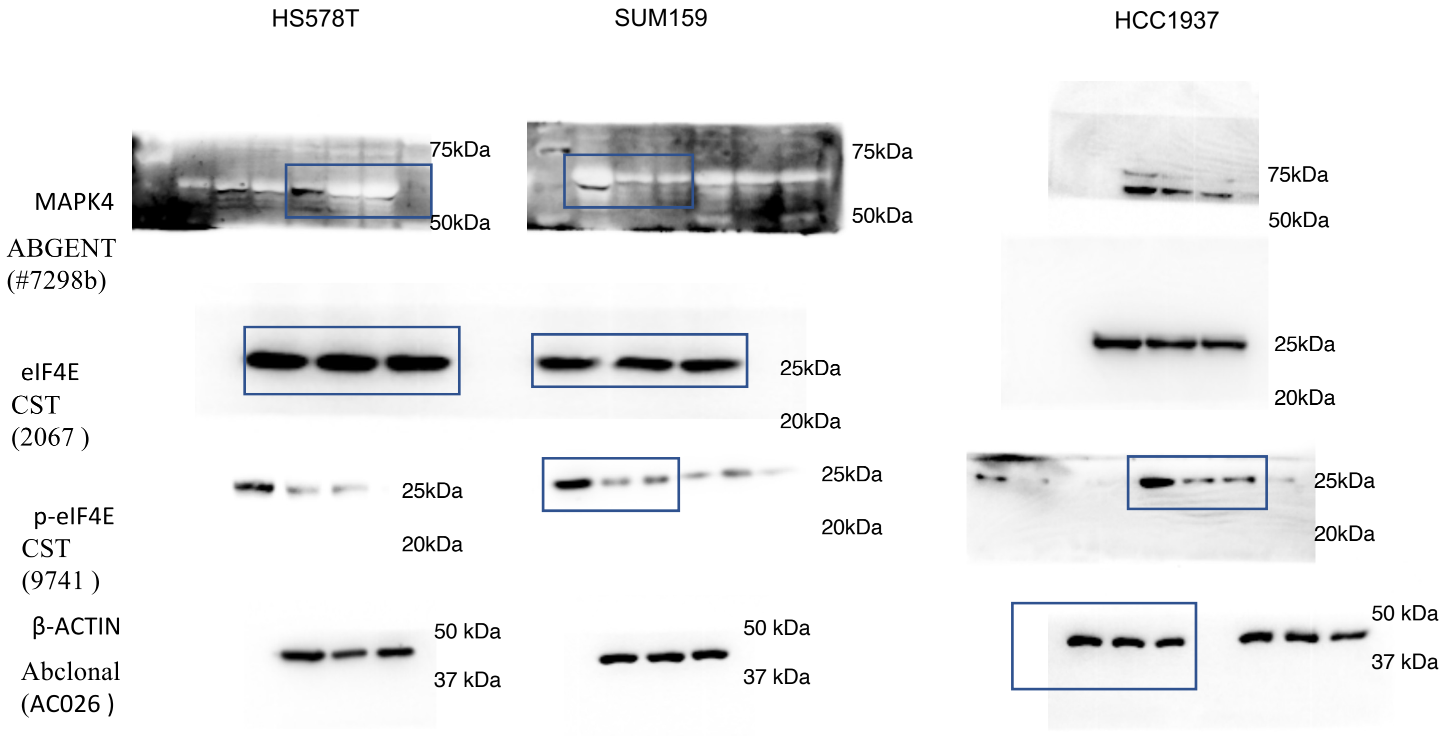

Figure S2 D

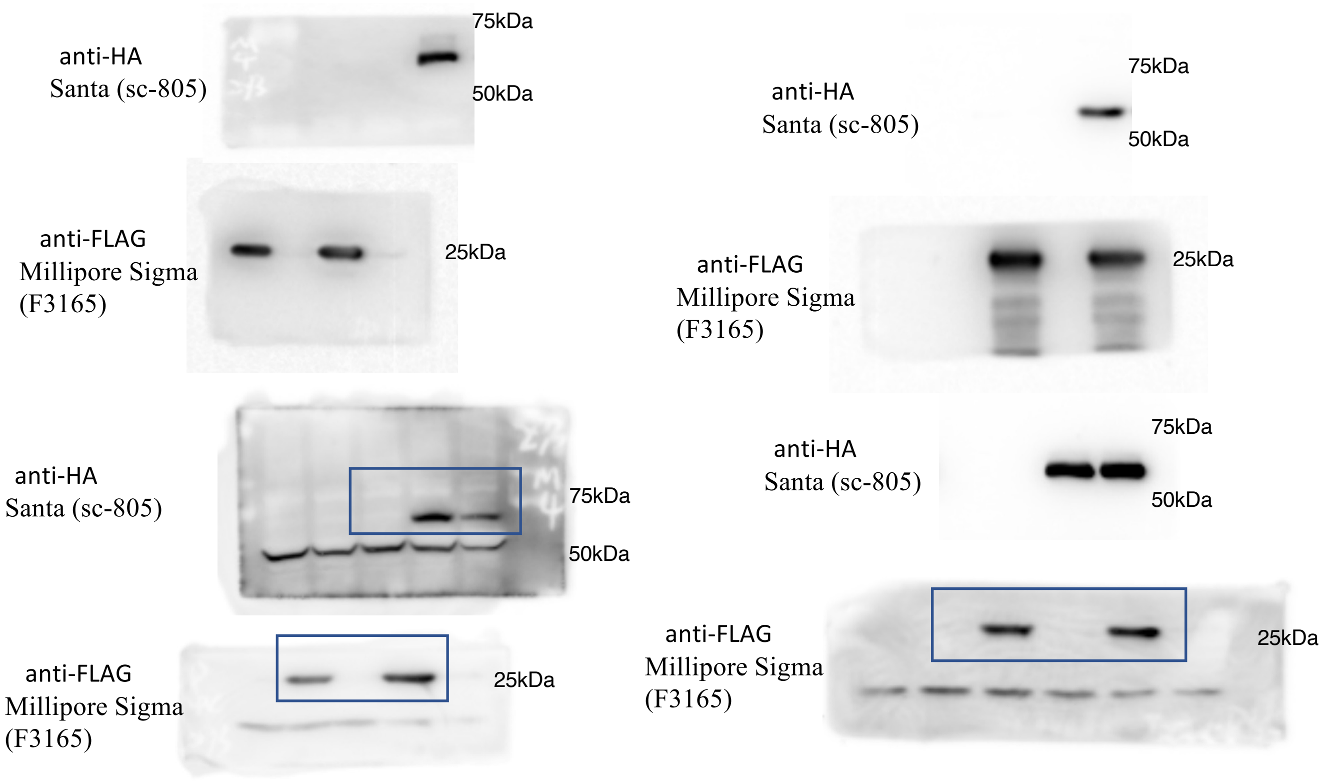

Figure S2 E

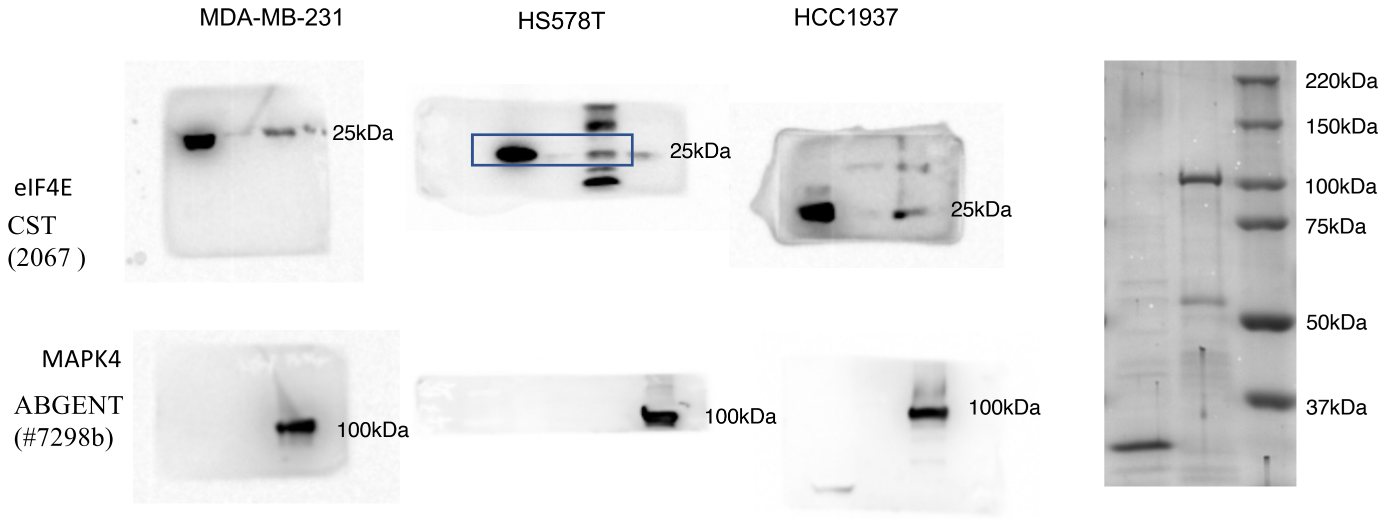

Figure S3A

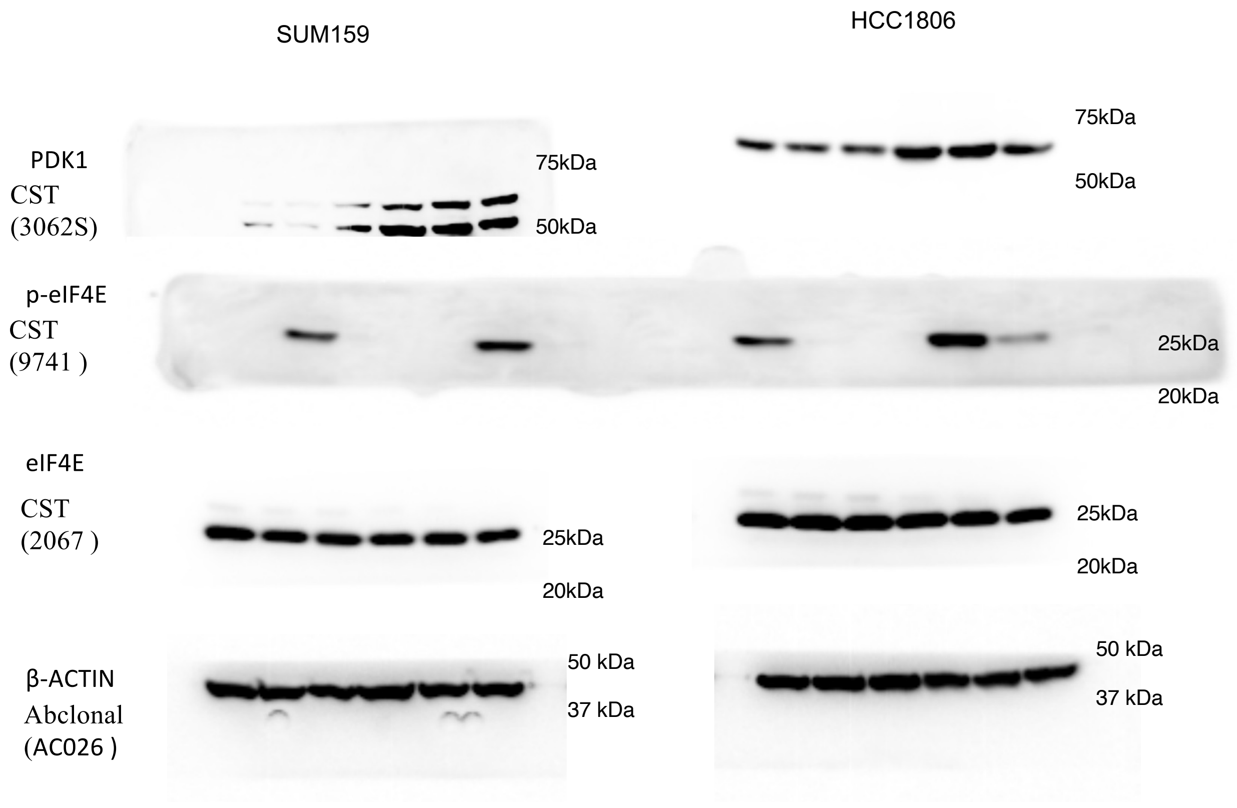

Figure S3B

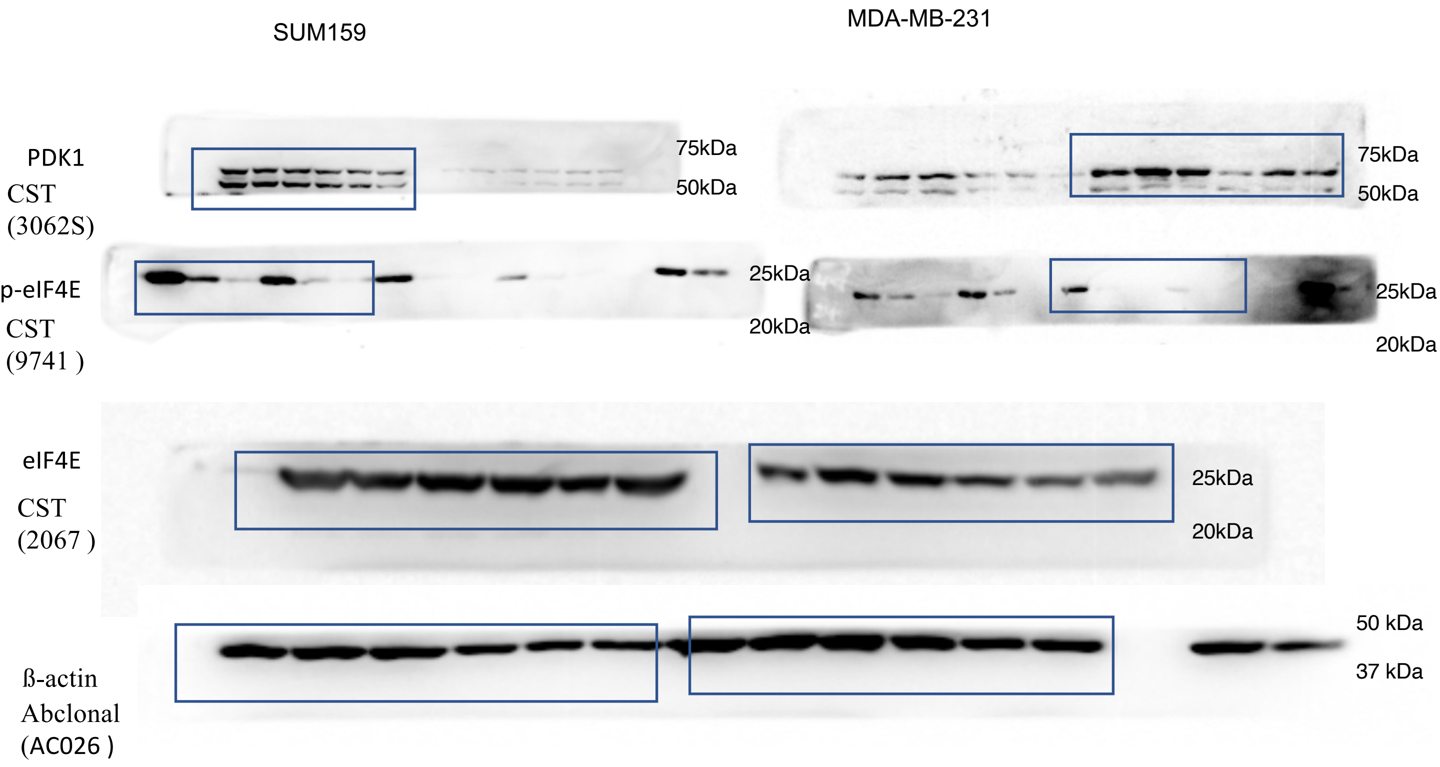

Figure S5

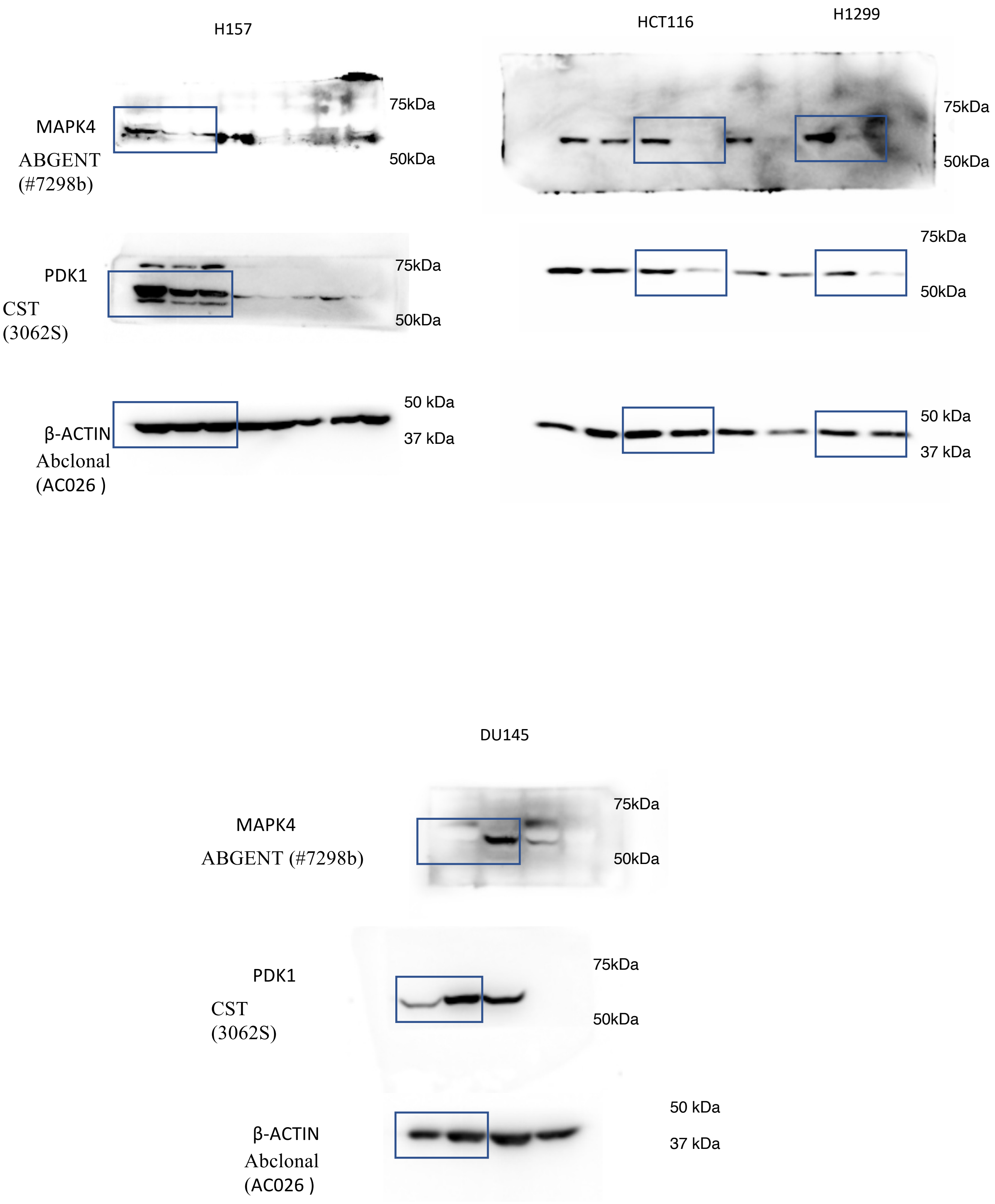
H157
HCT116
H1299
MAPK4
ABGENT
(#7298b)
75kDa
50kDa
PDK1
CST
(3062S)
75kDa
50kDa
β-ACTIN
Abclonal
(AC026)
50 kDa
37 kDa
DU145
MAPK4
ABGENT (#7298b)
75kDa
50kDa
PDK1
CST
(3062S)
75kDa
50kDa
β-ACTIN
Abclonal
(AC026)
50 kDa
37 kDa
